# Surface-redox sodium-ion storage in anatase titanium oxide

**DOI:** 10.1038/s41467-022-35617-3

**Published:** 2023-01-03

**Authors:** Qiulong Wei, Xiaoqing Chang, Danielle Butts, Ryan DeBlock, Kun Lan, Junbin Li, Dongliang Chao, Dong-Liang Peng, Bruce Dunn

**Affiliations:** 1 Department of Materials Science and Engineering, Fujian Key Laboratory of Surface and Interface Engineering for High Performance Materials, Xiamen Key Laboratory of High Performance Metals and Materials, College of Materials, Xiamen University, Xiamen, 361005 PR China; 2 Innovation Laboratory for Sciences and Technologies of Energy Materials of Fujian Province (IKKEM), Xiamen, 361005 PR China; 3 Department of Materials Science and Engineering, University of California Los Angeles, Los Angeles, CA 90095 USA; 4 Laboratory of Advanced Materials, Department of Chemistry, State Key Laboratory of Molecular Engineering of Polymers, iChEM (Collaborative Innovation Center of Chemistry for Energy Materials), Fudan University, Shanghai, 200433 PR China; 5 College of Chemistry and Chemical Engineering, Inner Mongolia University, Hohhot, 010070 PR China

**Keywords:** Batteries, Electrochemistry, Batteries

## Abstract

Sodium-ion storage technologies are promising candidates for large-scale grid systems due to the abundance and low cost of sodium. However, compared to well-understood lithium-ion storage mechanisms, sodium-ion storage remains relatively unexplored. Herein, we systematically determine the sodium-ion storage properties of anatase titanium dioxide (TiO_2_(A)). During the initial sodiation process, a thin surface layer (~3 to 5 nm) of crystalline TiO_2_(A) becomes amorphous but still undergoes Ti^4+^/Ti^3+^ redox reactions. A model explaining the role of the amorphous layer and the dependence of the specific capacity on the size of TiO_2_(A) nanoparticles is proposed. Amorphous nanoparticles of ~10 nm seem to be optimum in terms of achieving high specific capacity, on the order of 200 mAh g^−1^, at high charge/discharge rates. Kinetic studies of TiO_2_(A) nanoparticles indicate that sodium-ion storage is due to a surface-redox mechanism that is not dependent on nanoparticle size in contrast to the lithiation of TiO_2_(A) which is a diffusion-limited intercalation process. The surface-redox properties of TiO_2_(A) result in excellent rate capability, cycling stability and low overpotentials. Moreover, tailoring the surface-redox mechanism enables thick electrodes of TiO_2_(A) to retain high rate properties, and represents a promising direction for high-power sodium-ion storage.

## Abstract (web summary)

Sodium ion storage remains relatively unexplored in comparison with well-understood lithium ion storage mechanisms. Here, the authors systematically investigate the surface-redox sodium ion storage properties of anatase titanium dioxide, which delivers excellent rate capability, cycling stability and low overpotentials.

## Introduction

Pseudocapacitive materials store charge through Faradaic reactions at rapid rates, offering a route for achieving both high energy and power densities^1–3^. In addition to well-known H^+^ and Li^+^ systems, pseudocapacitive materials can also reversibly store charge in “beyond-lithium” systems including Na^+^, K^+^, and Zn^2+ ^^4–10^. Pseudocapacitive storage is especially promising for high-rate sodium-ion storage which could enable technologies such as large-scale grid storage^11–14^.

Pseudocapacitive mechanisms include surface-redox pseudocapacitance and intercalation pseudocapacitance^1,15^. Surface-redox pseudocapacitance occurs when Faradaic charge transfer processes take place at or near the surface of a material^3,4^. Intercalation pseudocapacitance is a bulk phenomenon, similar to battery-type intercalation. Both pseudocapacitive responses are characterized by capacitor-like electrochemical signatures and their kinetic behavior is not dominated by diffusion controlled processes^16–18^. Interestingly, certain intercalation materials for lithium-ion batteries demonstrate pseudocapacitive behavior when their particle size is reduced to several nanometers (termed extrinsic pseudocapacitive materials)^1,3,4,19^. One of these materials is anatase TiO_2_ (TiO_2_(A))^20,21^. TiO_2_(A) has a tetragonal crystal structure with empty octahedral sites that reversibly accommodate Li^+^ intercalation and extraction^22^. Briefly, Li^+^ intercalation into TiO_2_(A) occurs in three steps: solid solution (formation of *α*-Li_*x*_TiO_2_), phase transformation from *α*-Li_*x*_TiO_2_ to *β*-Li_*x*_TiO_2_ (with a plateau at ~1.75 V *vs*. Li^+^/Li), and interfacial storage (from 1.7 to 1 V *vs*. Li^+^/Li)^21^. Research groups report that the specific capacity of TiO_2_(A) for lithium-ion storage is size-dependent arising from the slow Li^+^ diffusion in the *β*-Li_*x*_TiO_2_^22,23^. In addition, when the particle sizes of TiO_2_(A) were reduced to ~7 nm, surface-controlled reactions due to the “nanosize effect” led to enhanced rate capability^20^.

TiO_2_(A) also shows promising sodium-ion storage performance^24–34^, however, results regarding the sodium-ion storage mechanism of TiO_2_(A) are inconsistent. Previous literature showed that ex-situ^24,25^ and in-situ^29^ X-ray diffraction (XRD) patterns display unchanged diffraction peaks of crystalline TiO_2_(A) in both sodiated and desodiated states. In contrast, other publications based on the observation of small sized TiO_2_(A) nanoparticles (NPs), have indicated a complete loss of crystallinity after initial sodiation to 0.01 V *vs*. Na^+^/Na with the amorphous state maintained during subsequent cycles^30^. These two different responses lead to two conflicting explanations: the former proposed an intercalation mechanism without phase changes^24,25,29^, while the latter indicated an irreversible transition from crystalline TiO_2_(A) into an amorphous phase after the initial sodiation^30^. Although previous results showed different specific capacities of various TiO_2_(A) nanomaterials^24–34^, the above two explanations do not give rise to a consistent model which details the relationships among the capacities, particle sizes, and charge storage mechanism. Additionally, the sodium-ion storage of TiO_2_(A) anodes were considered to have pseudocapacitive responses^30^, but it was not determined whether the pseudocapacitive response is extrinsic (caused by “nanosize effect”) or intrinsic (inherent capacitor-like charge kinetics)^1,3^. If we are to understand the sodium-ion storage mechanism for TiO_2_(A), the above results must be reconciled and a consistent model needs to be proposed.

In this work, we systematically investigate the electrochemical properties of TiO_2_(A) with particle sizes ranging from 5 to 100 nm to better understand the sodium-ion storage mechanism. A combination of methods including ex-situ/in-situ XRD, ex-situ transmission electron microscopy (TEM), and ex-situ X-ray photoelectron spectroscopy (XPS) were used to characterize different-sized TiO_2_(A) nanoparticles (NPs). These results demonstrate that only a thin surface layer (~3 to 5 nm) of crystalline TiO_2_(A) NPs becomes amorphous upon the initial sodiation process and remains amorphous in subsequent cycles. Based on our finding, a surface-dependent charge storage model is proposed, that details the relationship between particle size and specific capacity, and reconciles the inconsistencies in the literature reported for bulk and nanomaterials. When the particle size is significantly larger than the amorphous surface layer, the XRD patterns remain unchanged^24,25^, while the XRD patterns show the disappearance of peaks when the particle size is less than 10 nm^30^. The kinetic analysis based on cyclic voltammetry (CV) measurements indicate an intrinsic surface-redox reaction mechanism, which is size-independent and exhibits capacitor-like kinetics. Electrochemically, we demonstrate high-rate capability and excellent cycling stability when TiO_2_ is used as a pseudocapacitive, sodium-ion storage anode.

## Results

### Characterization of TiO_2_(A) NPs

XRD patterns of the different-sized TiO_2_ NPs are shown in Supplementary Fig. 1a. All diffraction peaks correlate to the anatase phase without any impurities. The average crystallite sizes of the TiO_2_-NPs were calculated according to the Scherrer formula^20^. The calculated crystallite sizes of TiO_2_ NPs and the corresponding Brunauer-Emmett-Teller (BET) surface areas of different TiO_2_ are listed in Supplementary Table 1. Assuming the NPs are spherical, the relationship between theoretical surface area (*S*) and particle sizes (*d*) follows *S* = *dρ*/6^20^, where the density (*ρ*) of TiO_2_(A) is 3.82 g cm^−3^. These calculated results (Supplementary Table 1) are consistent with the particle size observed by TEM (Supplementary Fig. 2).

### Influence of particle size on sodium-ion storage of TiO_2_(A)

**Fig. 1:**
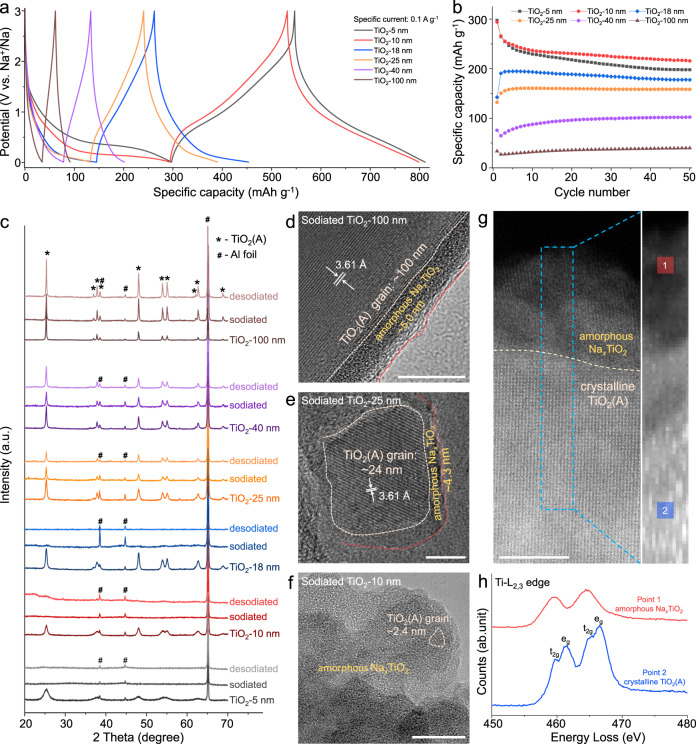
Structure and charge storage properties of various TiO_2_(A) NPs. The initial sodiation and desodiation curves (**a**) and cycling performance at 0.1 A g^−1^ (**b**) of different TiO_2_ NPs, respectively. **c** Ex-situ XRD patterns of different TiO_2_ NPs. Below 18 nm the (de)sodiated NPs are X-ray amorphous, whereas larger ones (>18 nm) remain crystalline. The ex-situ HRTEM images of TiO_2_-100 nm (**d**), TiO_2_−25 nm (**e**), and TiO_2_−10 nm (**f**) at the fully sodiated state, showing the amorphous Na_*x*_TiO_2_ shell and crystalline TiO_2_(A) core structure. Scale bar: 10 nm. HAADF-STEM image (**g**) and EELS spectra of Ti-L_2,3_ (**h**) of sodiated TiO_2_−25 nm. Scale bar: 5 nm.

The electrochemical performance of different TiO_2_ NPs is obtained using half cells with sodium metal as a counter/reference electrode. An electrolyte of 1 M NaPF_6_ in diglyme was used as it promotes the formation of relatively thin SEI layers^30,31^. The specific capacity of TiO_2_ NPs is carefully calculated to exclude the contribution from the conductive carbon additives (the detailed calculation steps are described in “Methods” section). Figure 1a shows the initial sodiation and desodiation curves of the six TiO_2_ NPs at the specific current of 0.1 A g^−1^ (corresponding to the current density of 0.15 mA cm^−2^) in the potential window of 0.01–3 V vs. Na^+^/Na. The first cycle sodiation/desodiation capacities for the various NPs are listed in Table 1. The specific capacity of TiO_2_-NPs is size-dependent with smaller sizes exhibiting higher capacity.

**Table 1:** The specific surface area and sodium-ion storage performance of different TiO_2_ NPs at 0.1 A g^−1^ (0.15 mA cm^−2^)

Sample	Specific surface area (m^2^ g^−1^)	Initial sodiation/desodiation capacities (mAh g^−1^)	Second sodiation capacity (mAh g^−1^)
TiO_2_-5 nm	303	297/250	265
TiO_2_-10 nm	133	294/235	265
TiO_2_-18 nm	80	142/113	191
TiO_2_-25 nm	57	132/105	151
TiO_2_-40 nm	40	76/50	65
TiO_2_-100 nm	11	34/23	27

The cycling performance and the corresponding discharge-charge curves of the TiO_2_ NPs at 0.1 A g^−1^ (0.15 mA cm^−2^) are shown in Fig. 1b and Supplementary Fig. S3. TiO_2_-5 nm and TiO_2_-10 nm deliver a reversible specific capacity of ~265 mAh g^−1^, corresponding to Na_*x*_TiO_2_ (*x* ≈ 0.8). Both materials show capacity fading during the initial cycles, but after ~10 cycles it is evident that TiO_2_-10 nm exhibits superior cycling stability compared with that of TiO_2_-5 nm. In contrast, the TiO_2_-18, 25, 40, and 100 nm anodes show an increase in specific capacity with repeated cycling.

Ex-situ XRD patterns of different-size TiO_2_ NPs are used to characterize the structural features during sodiation and desodiation (Fig. 1c). A complete lack of crystallinity was observed for sodiated and desodiated TiO_2_-5, 10, and 18 nm (the peaks at 38.5°, 44.7° and 65.2° are from Al foil current collector), while the diffraction peaks are broadened but unchanged for the sodiated and desodiated TiO_2_−25, 40, and 100 nm. The loss of crystalline structure is not recovered in the subsequent desodiation process.

Ex-situ high-resolution TEM (HRTEM) was used to further investigate the amorphous transitions. The sodiated TiO_2_-100 nm (Fig. 1d) and TiO_2_-25 nm (Fig. 1e) display the amorphous Na_*x*_TiO_2_ shell and crystalline TiO_2_(A) core structure with a shell thickness of ~5.0 and 4.3 nm, respectively. In comparison, the HRTEM image of sodiated TiO_2_-10 nm (Fig. 1f) displays a lack of long-range order with ultrafine TiO_2_(A) grains (~2 nm). Additionally, high-angle annular dark-field scanning transmission electron microscopy (HAADF-STEM) image of the sodiated TiO_2_-25 nm (Fig. 1g) confirms the amorphous surface layer and crystalline core. Electron energy loss spectroscopy (EELS) spectra of Ti-L_2,3_ edges (Fig. 1h) show that the crystalline core (point 2 in Fig. 1g) remains with the typical peaks of anatase TiO_2_, while, the peaks from surface amorphous Na_*x*_TiO_2_ (point 1 in Fig. 1g) indicate the reduction of surface Ti^4+^ after sodiation^35,36^. It should be noted that acid treatment has been used to remove SEI layers^37,38^. This treatment does not influence the phase and surface structure of TiO_2_(A) NPs (Supplementary Fig. 4), and after acid treatment, the surface amorphous layers are also observed (Supplementary Fig. 5). These ex-situ HRTEM results indicate that the amorphous layers are from the electrochemical sodiation process, consistent with the loss of crystallinity observed by ex-situ XRD (Fig. 1c).

**Fig. 2:**
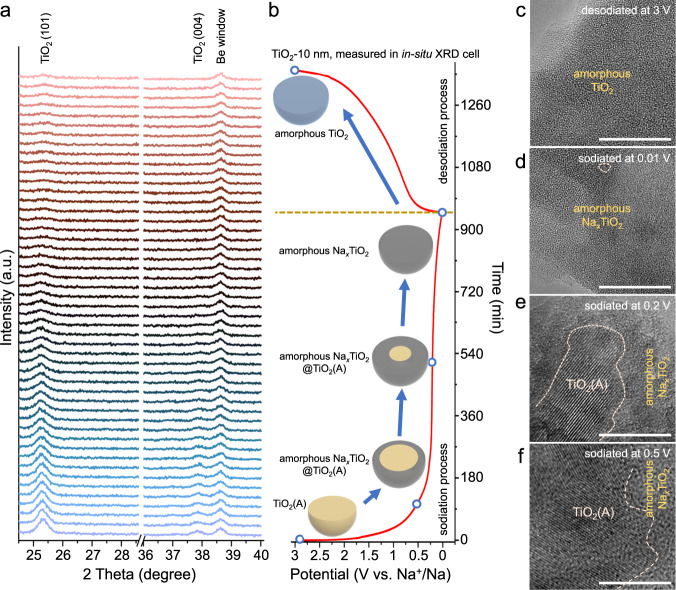
Structure evolution of TiO_2_-10 nm upon sodiation and desodiation. In-situ XRD patterns (**a**) and corresponding initial sodiation and desodiation curves at 0.03 A g^−1^ (**b**) of TiO_2_-10 nm. **c** Ex-situ HRTEM images of TiO_2_-10 nm desodiated at 3 V (**c**), and sodiated at 0.01 V (**d**), 0.2 V (**e**), and 0.5 V (**f**). The images indicate a continuous crystalline to amorphous transition at the particle surface which progresses inwards. Scale bar: 10 nm.

In-situ XRD was collected for two samples, TiO_2_-10 nm and TiO_2_-100 nm, to assess the structural changes associated with sodiation and desodiation. Figure 2a shows the in-situ XRD patterns of TiO_2_-10 nm and the corresponding initial sodiation and desodiation curves (Fig. 2b) at 0.03 A g^−1^ (0.045 mA cm^−2^). The first sodiation curve of the TiO_2_-10 nm anode displays a plateau at ~0.25 V *vs*. Na^+^/Na. At this potential, the TiO_2_(101) and (004) peaks do not shift upon initial sodiation, but broaden and disappear with additional sodiation. This series of XRD scans indicates that the TiO_2_ transitions from a crystalline to an amorphous state upon the sodiation plateau. In the subsequent desodiation process, the TiO_2_ remains in the amorphous state, and the galvanostatic charge profile displays a curved slope, which is typical of pseudocapacitive charge storage behavior^1,3^. Additionally, ex-situ HRTEM images of TiO_2_-10 nm at different sodiation states (Fig. 2c–f) indicate a continuous crystalline to amorphous transition at the particle surface which progresses inward (schematically shown in Fig. 2b).

In contrast, the TiO_2_-100 nm shows a rather limited sodiation characteristic, with no evidence of a plateau and with a curved galvanostatic discharge-charge profile (Fig. 1a and Supplementary Fig. 3f). Moreover, the in-situ XRD patterns exhibit no change in the crystalline phase of anatase during six cycles (Supplementary Fig. 6). The two different results for TiO_2_-10 nm and TiO_2_-100 nm indicate that the particle sizes have a significant influence on the electrochemical sodium-ion storage which may explain the discrepancies of XRD peaks in previously reported data^24,25,29,30^. This finding is consistent with the ex-situ XRD patterns of different-size TiO_2_ NPs (Fig. 1c).

Ex-situ Ti 2p XPS spectra of TiO_2_-10 nm and TiO_2_-100 nm (Supplementary Figs. 7, 8 and Supplementary Table 2) show the reversible redox of Ti^4+^/Ti^3+^ during sodiation and desodiation. It is significant to note that both materials exhibit the presence of the similar amorphous surface composition (Na_*x*_TiO_2_ with *x* = 0.8). Moreover, neither metallic Ti nor Na_2_O were observed in the ex-situ XPS spectrum, ex-situ XRD, or TEM results. This is consistent with the findings of Siebert et al.^39^ who demonstrated the reversible Ti^4+^/Ti^3+^ redox reaction through *operando* X-ray absorption near-edge structure spectroscopy (XANES) of the Ti K-edge. Additionally, extended X-ray absorption fine structure (EXAFS) indicated the coexistence of Ti^4+^–O and Ti^3+^–O bonds after sodiation and the reversible shrinkage of Ti–O bonds after desodiation^30^. These results confirm that the sodium-ion storage of TiO_2_(A) is not a conversion reaction and is exclusively based on the Ti^4+^/Ti^3+^ redox couple.

**Fig. 3:**
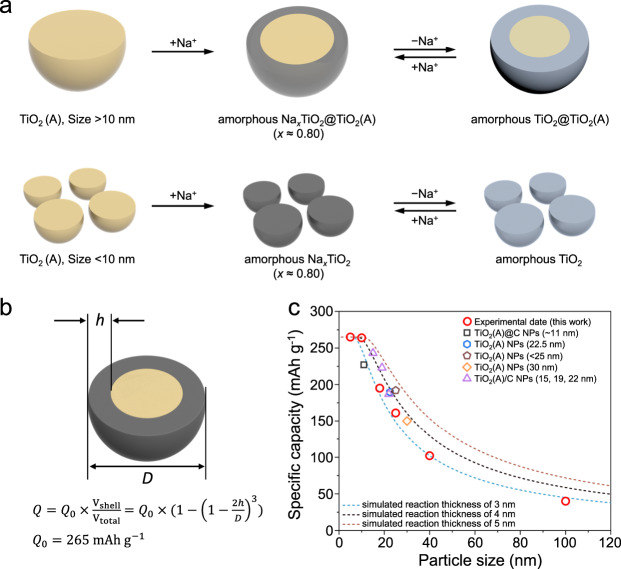
Overview of the sodium-ion storage mechanism of TiO_2_(A). **a** For particles >10 nm, only a thin surface layer of crystalline TiO_2_(A) is amorphized at the initial sodiation process, producing the sodiated amorphous composition of Na_*x*_TiO_2_ (*x* = 0.80). For particles <10 nm, they totally tend into amorphous state. The materials remain in the amorphous state with reversible redox of Ti^4+^/Ti^3+^ in the following cycles. **b, c** A model fitting the particle size, delivered specific capacity and reaction thickness of the TiO_2_ NPs. The sodiated surface layer produces a high capacity (*Q*_0_ = 265 mAh g^−1^), but the specific capacity of the entire TiO_2_ particle depends on the proportion of the reaction thickness (*h*) to the total spherical diameter (*D*). The measured reversible specific capacity of different-sized TiO_2_ (this work and refs. 27,30,32–34) fit well with the reaction thickness of 3–5 nm, which is consistent with the experimental observations. The limited surface reaction thickness is responsible for the particle size dependence of the specific capacity.

The sodium-ion storage mechanism of TiO_2_(A) is summarized schematically in Fig. 3a. The initial sodiation of TiO_2_(A) involves a gradual surface amorphization process, but importantly, the reaction depth is only ~3 to 5 nm. The surface-sodiated compositions of different-sized NPs are consistent, Na_0.80_TiO_2_. After the initial sodiation, the following charge/discharge cycles rely on the redox properties of the surface Na_*x*_TiO_2_. This mechanism explains the different observations for in-situ/ex-situ XRD patterns (Fig. 1c, Fig. 2a and Supplementary Fig. 6) and previous reports^24,25,29,30^. Since the amorphization is on the order of ~3 to 5 nm, the TiO_2_-10 nm completely transforms into an amorphous material leading to the disappearance of XRD peaks (Figs. 1c and 2a) and the long initial sodiation plateau (Fig. 1a). The particle cores of the TiO_2_ NPs over 25 nm, however, remain largely intact, resulting in well-defined XRD peaks throughout cycling (Fig. 1c).

Because of the limited surface-reaction thickness, the specific capacity is largely dependent on particle size. TiO_2_ NPs deliver maximum capacity when the particle size is below 10 nm and the specific capacity decreases rapidly when the NP size is increased. Here, a mathematic model is proposed (Fig. 3b) based on a core-shell structure of the spherical particle. Assuming the reacted surface shell delivers the same capacity (*Q*_0_) for each material, the specific capacity of a given TiO_2_ particle is dependent on the proportion of the reaction thickness (*h*) to the total spherical diameter (*D*). Figure 3c shows the simulated curves based on the relations among the reaction thickness (*h*), particle size (*D*), and the obtained specific capacity (*Q*). The measured reversible specific capacity (Supplementary Table 3) for different TiO_2_ NPs, previously reported TiO_2_(A) nanoparticles^27,30,32,33^ and olive-shaped TiO_2_(A) NPs/carbon composites (TOC) with various sizes (~15, 19 and 22 nm)^34^ fit well for an assumed reaction thickness between 3 and 5 nm. This response is consistent with experimental observations (Fig. 1d–f).

### Sodium-ion storage processes for TiO_2_

**Fig. 4:**
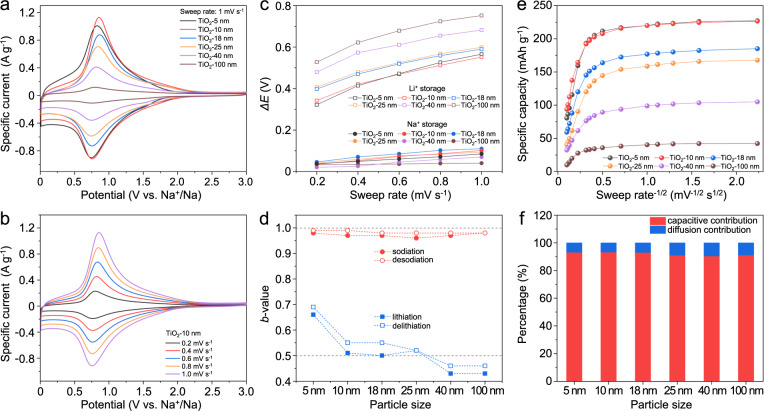
Charge storage properties of TiO_2_ for lithium and sodium ions. **a** CV curves of the TiO_2_ NPs at the sweep rate of 1.0 mV s^−1^ showing small voltage offset between redox peaks. **b** CV curves of TiO_2_-10 nm at sweep rates of 0.2–1.0 mV s^−1^, indicating slight peak shifts with increasing sweep rates. **c** The potential gap between the cathodic and anodic peaks ($\varDelta E$) as a function of particle size and sweep rate for Na^+^ and Li^+^. A much smaller potential offset for sodium-ion storage is observed. **d** The fitted *b*-values of the (de)sodiation and (de)lithiation peaks of TiO_2_ NPs, where the ${b}_{{{{{{{\rm{Na}}}}}}}^{+}}$≈1 indicates a capacitor-like process. **e** Specific capacity vs. *ν*^−1/2^ curves of the TiO_2_ NPs. **f** The capacitive and diffusion contributions for the TiO_2_ NPs at the sweep rate of 1.0 mV s^−1^.

To achieve better understanding of the sodium-ion storage process for the TiO_2_ NPs, a comparison was made with that of lithium-ion storage for the same range of TiO_2_ NPs. Results for the full range of TiO_2_ NPs for sodium-ion storage are presented in Supplementary Section 3, Figs. 9–15, while the corresponding TiO_2_ NPs for lithium-ion storage are displayed in Supplementary Section 4, Figs. 16–19. Figure 4a shows that the CV curves for all TiO_2_ NPs (at 1 mV s^-1^) exhibit similar redox peaks centered at ~0.75 V *vs*. Na^+^/Na, corresponding to the redox of Ti^4+^/Ti^3+^ from the amorphous TiO_2_ layer (as indicated by the ex-situ XPS, Supplementary Fig. 8). With the increase in sweep rates, the TiO_2_ NPs show slight peak shifts (Fig. 4b and Supplementary Fig. 9). Even over narrow potential ranges, the TiO_2_ anodes show the same redox peaks, indicating the highly reversible surface-redox reaction (Supplementary Fig. 10). The potential gap between the cathodic and anodic peaks ($\varDelta E$) for both sodium and lithium are shown as a function of particle size and sweep rate in Fig. 4c. At the sweep rate of 0.2 mV s^−1^, the ${\varDelta E}_{{{{{{{\rm{Na}}}}}}}^{+}}$ of all NPs are <0.045 V, much smaller than those of ${\varDelta E}_{{{{{{{\rm{Li}}}}}}}^{+}}$ (>0.32 V). With the increase of sweep rates, the ${\varDelta E}_{{{{{{{\rm{Na}}}}}}}^{+}}$ (<0.11 V at 1 mV s^−1^) are much lower than that of ${\varDelta E}_{{{{{{{\rm{Li}}}}}}}^{+}}$ (>0.55 V at 1 mV s^−1^) as well. The smaller potential offsets are typical for pseudocapacitive charge storage processes and very different from those of battery-type intercalation materials^1,3^.

The CV measurements were also used to characterize the kinetics of the charge storage process. In this analysis, the peak current $({i}_{p})$ follows the power-law shown in Eq. 1^19^:

1 $${i}_{{{{{\rm{p}}}}}}=a{\nu }^{b}$$

where the *b*-value of 0.5 refers to a semi-infinite diffusion process, and *b* = 1 indicates a surface-controlled or capacitor-like kinetics. The *b*-values for the sodium-ion and lithium-ion storage are calculated in Supplementary Figs. 11 and 17, respectively. For lithium-ion storage of TiO_2_(A), the material is a well-known insertion-type anode and ${b}_{{{{{{{\rm{Li}}}}}}}^{+}}$ ≈ 0.5 (Fig. 4d). When the TiO_2_-NP size is reduced into the nanometer range, the value of ${b}_{{{{{{{\rm{Li}}}}}}}^{+}}$ for TiO_2_-5 nm is increased to 0.69, consistent with previous reports^20^. In contrast, the ${b}_{{{{{{{\rm{Na}}}}}}}^{+}}$ values of all the TiO_2_ NPs are very close to 1, signifying surface-controlled kinetics (Fig. 4d).

To further explore the nature of diffusion-controlled and capacitive charge storage behaviors, the Trasatti analysis (Eq. 2) method was used^13^.

2 $$Q\left(\upsilon \right)={Q}_{{{{{{{\rm{capacitive}}}}}}}}+\alpha ({\upsilon }^{-1/2})$$

where *α* is a constant, and $Q(\upsilon )$ refers to the measured capacity, ${Q}_{{{{{{\rm{capacitive}}}}}}}$ is capacitive charge storage, and$\,{Q}_{{{{{{\rm{total}}}}}}}$ is the total amount of charge storage. For the sodium-ion storage of TiO_2_ NPs, the capacity is relatively independent at sweep rates below 4 mV s^−1^ (Fig. 4e), suggesting a capacitive type of process. In contrast, the capacities measured for lithium-ion storage tend to show a linear dependence on *ν*^−1/2^ which is consistent with a diffusion-controlled mechanism (Supplementary Fig. 18b).

A qualitative indication of the charge storage process can be obtained by separating the current response (*i*) into two contributions, namely capacitive (*k*_1_*ν*) and diffusion-controlled (*k*_2_*ν*^1/2^) (Eq. 3)^1^.

3 $$i\left(\upsilon \right)={k}_{1}\upsilon+{k}_{2}{\upsilon }^{1/2}$$

Supplementary Fig. 12 shows that the current arises almost exclusively from a capacitive contribution for all TiO_2_ NPs, at the sweep rate of 1 mV s^−1^. That is, the total capacity is dominated by capacitive contributions (over 90%, Fig. 4f).

In a confirming experiment, a TiO_2_−10 nm thin-film electrode (100 μg cm^−2^) was prepared without any conductive carbon additive or binders^16^. In this way, the influence of these other additives was eliminated. The CV curves for this thin-film electrode (Supplementary Fig. 13) and the corresponding *b* > 0.9 from Eq. 1 show the same charge storage behavior as the electrodes containing binder and conductive additive. In addition, the electrochemical behaviors of different TiO_2_ NPs measured in ester-based electrolyte (Supplementary Fig. 14) are consistent with the results of ether-based electrolyte. These results support the contention that the sodium-ion charge storage process for TiO_2_ NPs is the intrinsic surface-redox mechanism that arises from the electrochemically formed amorphous surface layer, and is independent of the electrolyte system used in the experiments.

**Fig. 5:**
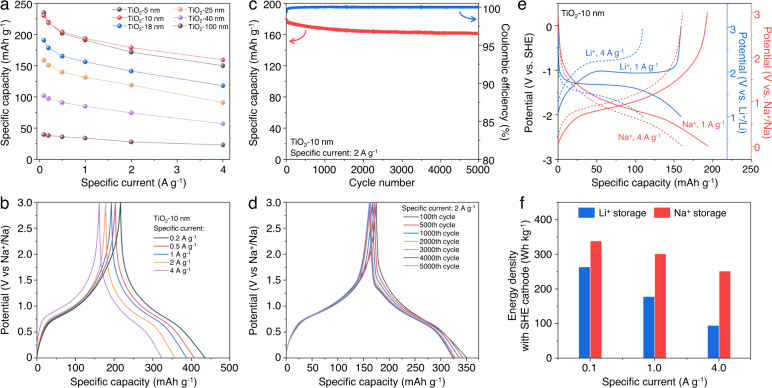
Sodium-ion storage properties of TiO_2_ NPs. **a** Rate capability of the different TiO_2_ NPs. **b** Charge-discharge curves of the TiO_2_-10 nm at different specific currents, from 0.2 to 4 A g^−1^. Extended cycling (**c**) and related charge-discharge curves at various cycles (**d**) for the TiO_2_-10 nm at 2 A g^−1^. Excellent stability for 5000 cycles is shown. **e** The charge and discharge curves of the TiO_2_-10 nm for Li^+^ and Na^+^ storage *vs*. SHE. **f** The calculated average energy density for Li^+^ and Na^+^ storage for 10 nm TiO_2_ NPs. The calculation is based on using SHE as a hypothetical cathode. The higher energy density for Na^+^ storage at high power is very appealing for device applications.

The rate capabilities for sodium-ion storage for the various TiO_2_ NPs are shown in Fig. 5a. The TiO_2_−10 nm material, in particular, shows good specific capacity at high-rates, with values of nearly 200 mAh g^−1^ at 1 A g^−1^ (1.5 mA cm^−2^). Even at 4 A g^−1^ (6 mA cm^−2^) the material stores nearly 160 mAh g^−1^. The galvanostatic charge-discharge curves show little change in their profiles with increasing current density (Fig. 5b). Moreover, the TiO_2_-10 nm anode exhibits excellent fast‑charging and high‑rate performance when it is measured under fixed charging or discharging rates (Supplementary Fig. 15). The TiO_2_-10 nm also shows stable long-term cycling performance (Fig. 5c): the capacity retention is 90.0% after 5000 cycles at a specific current of 2 A g^−1^ (3.0 mA cm^−2^). During long-term cycling, the charge-discharge curves overlap well (Fig. 5d) without an increase in overpotential, indicating sodium-ion storage in TiO_2_ is highly reversible and stable.

An anode material with high specific capacity and a low operating potential is beneficial to obtaining high energy density in a full cell. The operating potential of TiO_2_ for sodium-ion storage (~0.75 V *vs*. Na^+^/Na, that is −1.96 V vs. standard hydrogen electrode (SHE)) is much lower than that of lithium-ion storage (~1.75 V vs. Li^+^/Li, that is −1.29 V vs. SHE) (Fig. 5e). To numerically compare the energy density of the TiO_2_ for both lithium-ion and sodium-ion storage, we calculated the energy density by using the SHE as a cathode reference, according to the methods suggested by Cao et al.^40^. By this methodology, the energy densities of TiO_2_-10 nm for sodium-ion storage (300 Wh kg^−1^) are higher than that of lithium-ion storage (176 Wh kg^−1^) (Fig. 5f), at the high specific current of 1 A g^−1^ (1.5 mA cm^−2^). Moreover, at a higher specific current (4 A g^−1^, 6.0 mA cm^−2^), the energy density of the TiO_2_-10 nm for sodium-ion storage exhibits much higher values than that for lithium-ion storage (250 *vs*. 93 Wh kg^−1^).

**Fig. 6:**
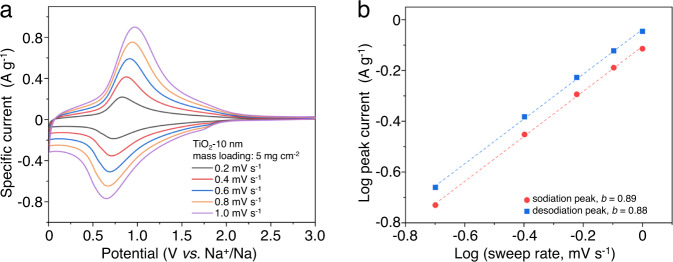
High-mass loading performance of TiO_2_ NPs. The small voltage offset in CV curves (**a**) and the *b*-value analysis (**b**) of the TiO_2_-10 nm for 5 mg cm^−2^ mass loading. The results indicate that surface-controlled kinetics also occur in thick electrodes.

Thick-film electrodes of TiO_2_-10 nm at a mass loading of 5 mg cm^-2^ were prepared in order to determine whether the greater mass would influence charge storage processes^41^. This thick electrode displays a similar CV response at varying sweep rates to those of 1.5 mg cm^−2^ (compare Fig. 6a with Fig. 4b). At this high-mass loading level, the *b*-values (Fig. 6b) determined from peak cathodic/anodic currents were on the order of 0.9, once again indicating surface-controlled kinetics. Taken together, these results for high mass loadings exhibit several pseudocapacitive signatures and offer the prospect of achieving high energy and high power, appealing features for practical applications.

## Discussion

A combination of in-situ and ex-situ characterization has been used to determine the sodium-ion storage mechanism of TiO_2_(A) nanoparticles which range in size from 5 to 100 nm. The sodium-ion storage properties are attributed to the presence of surface layers of 3–5 nm thick. During the initial sodiation process, the surface layers of TiO_2_(A) become amorphous and subsequent Ti^4+^/Ti^3+^ redox reactions in the amorphous titanium oxide surface layer lead to significant levels of charge storage. A model detailing the relationship between particle size and specific capacity is able to reconcile prior results in the literature which showed a very strong dependence on particle size. The best electrochemical performance occurs with small particles whose size (~10 nm) is such that nearly the entire particle is amorphous. These particles are X-ray amorphous whereas larger ones (>10 nm) remain crystalline.

Kinetic studies exhibit several pseudocapacitive signatures as redox reactions occur at the surface and near surface regions. These properties include peak currents with linear dependence on sweep rate, charge storage which is relatively independent of sweep rate and a small voltage offset between oxidation and reduction reactions. The kinetics for sodium-ion storage in TiO_2_(A) are very different from those of lithium-ion processes as the surface-redox mechanism for sodium-ion storage is not limited by semi-infinite diffusion and exhibits excellent rate capability, cycle stability and low overpotentials. We find that the surface-redox mechanism is also active in thick electrodes, which is promising for practical applications.

## Methods

### TiO_2_ nanoparticles

The TiO_2_ (anatase) nanoparticles are purchased from US Research Nanomaterials, Inc., and used without additional treatment.

### Material characterizations

The powder X-ray diffraction (XRD) was characterized on Bruker-Axs X-ray diffractometer (Cu *Kα* radiation, λ = 1.5406 Å). In-situ XRD was measured by using a customized two-electrode cell, in which a piece of Be disk were used as the current collector and the X-ray transparent window. Brunauer-Emmet-Teller (BET) specific surface area was measured by nitrogen sorption isotherms (Micromeritics Tristar 3020) at 77 K after degassed under vacuum at 200 °C for over 6 h. Transmission electron microscopy (TEM) images were recorded by using Titan G2 60–300. The high-angle annular dark-field scanning transmission electron microscopy (HAADF-STEM) images and electron energy loss spectroscopy (EELS) spectra were obtained through double spherical aberration corrected transmission electron microscope (Titan Cubed Themis G2 300). For the powder samples, the TiO_2_ NPs were sonicated in alcohol and dropped onto the grid for TEM observation. For preparing the ex-situ TEM samples, the coin cells were disassembled in an Ar-filled glove box after being electrochemically cycled at different states, and the electrodes were washed with the diglyme solvent. Then, the TiO_2_ NPs were carefully scratched from the Al foil, sonicated in diglyme and dropped onto the grid for TEM observation. For preparing the ex-situ TEM sample with removed SEI layers, the electrodes were washed with the diglyme solvent. Then, the TiO_2_ NPs were carefully scratched from the Al foil and soaked in 0.1 M HCl solution for 1 h. After centrifuging, the washed sample was sonicated in alcohol and dropped onto the grid for TEM observation. XPS survey scan analyses were conducted on a Kratos Axis Supra using monochromatized Al Kα X-ray source (1486.7 eV). The XPS chamber was directly connected to the Ar-filled grove box to avoid air exposure during sample preparation and transfer. The coin cell was disassembled in the Ar-filled glove box after electrochemically cycled at different states, and the TiO_2_ NPs electrode was washed with the diglyme solvent. After drying, the anodes were transferred to the XPS chamber for testing. Spectra were charge corrected to the main line of the carbon 1*s* spectrum and set to a binding energy of 284.8 eV.

### Electrode preparation and electrochemical measurement

The TiO_2_ electrodes were made from an aqueous slurry consisting of 85 wt.% TiO_2_ NPs, 7 wt.% Ketjen black, 4 wt.% carboxyl methyl cellulose (CMC) and 4 wt.% styrene butadiene rubber (SBR). For sodium-ion storage measurements, the slurry was cast onto Al foil using doctor blading. For lithium-ion storage measurements, the slurry was cast onto Cu foil. The coated slurry was dried at 120 °C under vacuum for 12 h. The mass loading of different-sized TiO_2_ electrodes were controlled at ≈1.5 mg cm^−2^. To measure the electrochemical performance of TiO_2_-10 nm at very thick films, the loading was increased to 5 mg cm^−2^. Coin cells (CR2032) were assembled in an Ar-filled glove box. For sodium-ion storage measurements, the sodium metal disk was used as the counter and reference electrode, Celgard-2325 used as separator, and 1 M NaPF_6_ in diglyme used as electrolyte. For lithium-ion storage measurements, lithium metal disk was used as counter and reference electrode, Celgard-2325 as separator, 1 M LiPF_6_ in ethylene carbonate (EC)/dimethyl carbonate (DMC) with a volume ratio of 50:50 as the electrolyte. Electrochemical measurements were carried out by Bio-Logic VSP potentiostat. For sodium-ion storage, the potential window is set in 0.01–3 V *vs*. Na^+^/Na. For lithium-ion storage, the potential window is set in 1–3 V *vs*. Li^+^/Li. All the coin cells were placed in an incubator with a stationary temperature of 25 °C for electrochemical measurements.

### Calculation the specific capacity of TiO_2_ NPs

The composite electrodes consist of 85 wt.% TiO_2_ NPs, 7 wt.% carbon additive (Ketjen black) and 8 wt.% binders (CMC + SBR). The total capacity of electrode ${Q}_{{{{{{\rm{total}}}}}}}={Q}_{{{{{{{\rm{TiO}}}}}}}_{2}}+{Q}_{{{{{{\rm{carbon}}}}}}}={Q}_{{{{{\rm{S}}}}},{{{{{{\rm{TiO}}}}}}}_{2}}\times {m}_{{{{{{{\rm{TiO}}}}}}}_{2}}+{Q}_{{{{{\rm{S}}}}},{{{{{\rm{carbon}}}}}}}\times {m}_{{{{{{\rm{carbon}}}}}}}$. As a rigorous approach to isolate the capacity from the TiO_2_ alone, additional sodiation capacity from the conductive carbon additive is measured for removal. The reversible specific capacity of carbon additive (${Q}_{{{{{{\rm{S}}}}}},{{{{{\rm{carbon}}}}}}}$) is 124 mAh g^−1^ at 0.05 A g^−1^ in the potential range of 0.01–3 V vs. Na^+^/Na, through making an electrode only consisting of carbon additives and binders. Then, by removing the contribution from the carbon additives, the specific capacity of TiO_2_ (${Q}_{{{{{{\rm{S}}}}}},{{{{{{\rm{TiO}}}}}}}_{2}}$) is calculated according to the following Eqs. 4 and 5.

4 $${Q}_{S,{{{{{{\rm{TiO}}}}}}}_{2}}=({Q}_{{{{{{\rm{total}}}}}}}-124{{{{{\rm{mAh}}}}}}{{{{{{\rm{g}}}}}}}^{-1}\times {m}_{{{{{{\rm{carbon}}}}}}})/{m}_{{{{{{{\rm{TiO}}}}}}}_{2}}$$

5 $${Q}_{S,{{{{{{\rm{TiO}}}}}}}_{2}}=({Q}_{{{{{{\rm{total}}}}}}}-124{{{{{\rm{mAh}}}}}}{{{{{{\rm{g}}}}}}}^{-1}\times {0.07m}_{{{{{{\rm{tota}}}}}}l})/{0.85m}_{{{{{{\rm{total}}}}}}}$$

### Calculation the energy density of TiO_2_ versus SHE reference

The energy densities (*E*) of TiO_2_ anodes for both lithium-ion and sodium-ion storages were calculated by using the standard hydrogen electrode (SHE) as the hypothetical cathode^40^. The galvanostatic desodiation (or delithiation) curves were applied for integrating based on $E=\int {{{{{\rm{I}}}}}}{{{{{\rm{V}}}}}}{dt}$, while the potential (V) versus SHE was applied.

## Supplementary information

Supplementary Information

## Data Availability

The data that support the findings of this study are available within the paper and Supplementary Information. Additional relevant data are available from the corresponding author on request.

## References

[1] Choi C (2020). Achieving high energy density and high power density with *pseudocapacitive materials*. Nat. Rev. Mater..

[2] Simon P, Gogotsi Y (2020). Perspectives for electrochemical capacitors and related devices. Nat. Mater..

[3] Fleischmann S (2020). Pseudocapacitance: from fundamental understanding to high power energy storage materials. Chem. Rev..

[4] Wei Q, DeBlock RH, Butts DM, Choi C, Dunn B (2020). Pseudocapacitive vanadium-based materials toward high-rate sodium-ion storage. Energy Environ. Mater..

[5] Liu N (2020). Intercalation pseudocapacitive Zn^2+^ storage with hydrated vanadium dioxide toward ultrahigh rate performance. Adv. Mater..

[6] Dong L (2019). High-power and ultralong-life aqueous zinc-ion hybrid capacitors based on pseudocapacitive charge storage. Nano-Micro Lett..

[7] Xu F (2020). Ultrastable surface-dominated pseudocapacitive potassium storage enabled by edge-enriched n-doped porous carbon nanosheets. Angew. Chem. Int. Ed..

[8] Chang X (2021). Pseudocapacitive anode materials toward high-power sodium-ion capacitors. Batteries Supercaps.

[9] Meng C (2021). In situ and operando characterizations of 2D materials in electrochemical energy storage devices. Small Sci..

[10] Chao D (2020). Roadmap for advanced aqueous batteries: from design of materials to applications. Sci. Adv..

[11] Ding J, Hu W, Paek E, Mitlin D (2018). Review of hybrid ion capacitors: from aqueous to lithium to sodium. Chem. Rev..

[12] Park H, Kwon J, Choi H, Song T, Paik U (2017). Microstructural control of new intercalation layered titanoniobates with large and reversible d-spacing for easy Na^+^ ion uptake. Sci. Adv..

[13] Cook JB (2016). Mesoporous MoS_2_ as a transition metal dichalcogenide exhibiting pseudocapacitive Li and Na-ion charge storage. Adv. Energy Mater..

[14] Liu Y (2018). Confining SnS_2_ ultrathin nanosheets in hollow carbon nanostructures for efficient capacitive sodium storage. Joule.

[15] Lukatskaya MR, Dunn B, Gogotsi Y (2016). Multidimensional materials and device architectures for future hybrid energy storage. Nat. Commun..

[16] Augustyn V (2013). High-rate electrochemical energy storage through Li^+^ intercalation pseudocapacitance. Nat. Mater..

[17] Fleischmann S (2022). Continuous transition from double-layer to Faradaic charge storage in confined electrolytes. Nat. Energy.

[18] Augustyn V, Gogotsi Y (2017). 2D materials with nanoconfined fluids for electrochemical energy storage. Joule.

[19] Augustyn V, Simon P, Dunn B (2014). Pseudocapacitive oxide materials for high-rate electrochemical energy storage. Energy Environ. Sci..

[20] Wang J, Polleux J, Lim J, Dunn B (2007). Pseudocapacitive contributions to electrochemical energy storage in TiO_2_ (anatase) nanoparticles. J. Phys. Chem. C..

[21] Lou S (2019). Ti-based oxide anode materials for advanced electrochemical energy storage: lithium/sodium ion batteries and hybrid pseudocapacitors. Small.

[22] Belak AA, Wang Y, Van der Ven A (2012). Kinetics of anatase electrodes: the role of ordering, anisotropy, and shape memory effects. Chem. Mater..

[23] Jiang C (2007). Particle size dependence of the lithium storage capability and high rate performance of nanocrystalline anatase TiO_2_ electrode. J. Power Sources.

[24] Longoni G (2017). Shape-controlled TiO_2_ nanocrystals for Na-ion battery electrodes: the role of different exposed crystal facets on the electrochemical properties. Nano Lett..

[25] Kim K-T (2014). Anatase titania nanorods as an intercalation anode material for rechargeable sodium batteries. Nano Lett..

[26] Lan K (2019). Two-dimensional mesoporous heterostructure delivering superior pseudocapacitive sodium storage *via* bottom-up monomicelle assembly. J. Am. Chem. Soc..

[27] Wu L (2015). Unfolding the mechanism of sodium insertion in anatase TiO_2_ nanoparticles. Adv. Energy Mater..

[28] Le Z (2017). Pseudocapacitive sodium storage in mesoporous single-crystal-like TiO_2_–graphene nanocomposite enables high-performance sodium-ion capacitors. ACS Nano.

[29] Chen Z (2019). Spray-pyrolysis-assisted synthesis of yolk@shell anatase with rich oxygen vacancies for efficient sodium storage. J. Mater. Chem. A.

[30] Xu Z-L (2018). Engineering solid electrolyte interphase for pseudocapacitive anatase TiO_2_ anodes in sodium-ion batteries. Adv. Funct. Mater..

[31] Li K (2019). Evolution of the electrochemical interface in sodium ion batteries with ether electrolytes. Nat. Commun..

[32] Tahir MN (2016). Extraordinary performance of carbon-coated anatase TiO_2_ as sodium-ion anode. Adv. Energy Mater..

[33] Chen J (2016). Black Anatase titania with ultrafast sodium-storage performances stimulated by oxygen vacancies. ACS Appl. Mater. Interfaces.

[34] Chen J (2016). Size-tunable olive-like anatase TiO_2_ coated with carbon as superior anode for sodium-ion batteries. Small.

[35] Bi X (2021). Tuning oxygen vacancy content in TiO_2_ nanoparticles to enhance the photocatalytic performance. Chem. Eng. Sci..

[36] Lü X (2016). Conducting interface in oxide homojunction: understanding of superior properties in black TiO_2_. Nano Lett..

[37] Wu H (2012). Stable cycling of double-walled silicon nanotube battery anodes through solid–electrolyte interphase control. Nat. Nanotechnol..

[38] Liu N (2014). A pomegranate-inspired nanoscale design for large-volume-change lithium battery anodes. Nat. Nanotechnol..

[39] Siebert A (2021). Monitoring the sodiation mechanism of anatase TiO_2_ nanoparticle-based electrodes for sodium-ion batteries by operando XANES measurements. ACS Appl. Energy Mater..

[40] Cao Y, Li M, Lu J, Liu J, Amine K (2019). Bridging the academic and industrial metrics for next-generation practical batteries. Nat. Nanotechnol..

[41] Fan HJ (2019). Pseudocapacitor electrodes: regular pores matter. Joule.

