# Supplementary material for: Surface-redox sodium-ion storage in anatase titanium oxide
Source: Nat Commun. 2023 Jan 3;14:7. doi: 10.1038/s41467-022-35617-3 (PMC9810695; doi:10.1038/s41467-022-35617-3)
Supplement: Supplementary file 1 — Supplementary Information [file 41467_2022_35617_MOESM1_ESM.pdf]

Supplementary Information for

# Surface-Redox Sodium-Ion Storage in Anatase Titanium Oxide

Qiulong Wei[1,2,*], Xiaoqing Chang[1], Danielle Butts[3], Ryan DeBlock[3], Kun Lan[4,5], Junbin Li[1], Dongliang Chao[4], Dong-Liang Peng[1,2], and Bruce Dunn[3,*]

[1]Department of Materials Science and Engineering, Fujian Key Laboratory of Surface and Interface Engineering for High Performance Materials, Xiamen Key Laboratory of High Performance Metals and Materials, College of Materials, Xiamen University, Xiamen 361005, P. R. China
[2]Innovation Laboratory for Sciences and Technologies of Energy Materials of Fujian Province (IKKEM), Xiamen 361005, P. R. China
[3]Department of Materials Science and Engineering, University of California Los Angeles, Los Angeles, CA, 90095, USA
[4]Laboratory of Advanced Materials, Department of Chemistry, State Key Laboratory of Molecular Engineering of Polymers, iChEM (Collaborative Innovation Center of Chemistry for Energy Materials), Fudan University, Shanghai, 200433, P. R. China
[5]College of Chemistry and Chemical Engineering, Inner Mongolia University, Hohhot 010070, P. R. China.

*Correspondence and requests for materials should be addressed to:
Prof. Qiulong Wei, E-mail: qlwei@xmu.edu.cn
Prof. Bruce Dunn, E-mail: bdunn@ucla.edu

**Supplementary Section 1**. Materials Characterizations

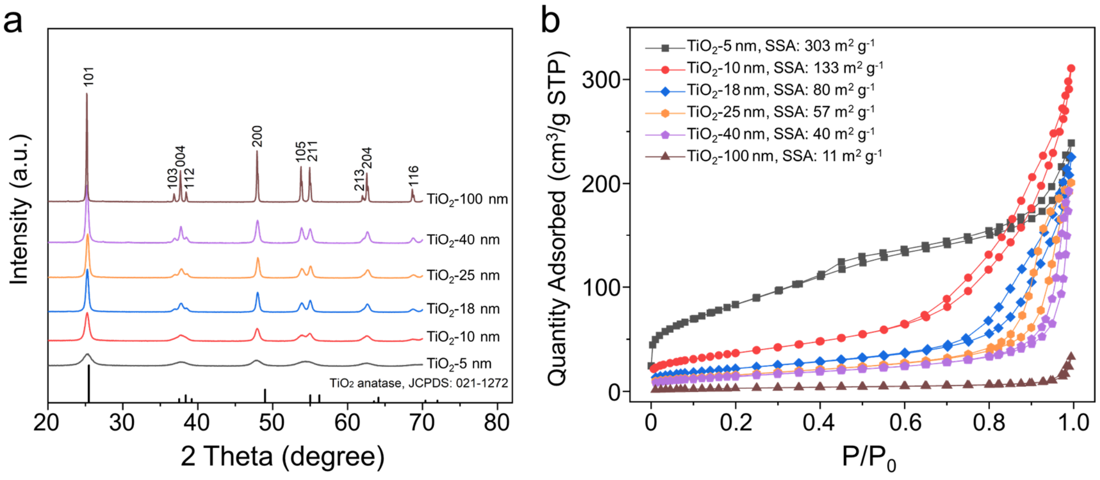


**Supplementary Figure 1.** (a) XRD patterns of $TiO_2$ nanoparticles (NPs) and (b) the $N_2$ adsorption-desorption isotherms of $TiO_2$ NPs. The average crystallite sizes of the $TiO_2$ NPs were calculated according to the Scherrer formula $d = 0.9\lambda_{Cu}/\omega$, where $d$ is the crystallite size, $\lambda_{Cu}$ = 1.5406 Å is the X-ray wavelength, and $\omega$ is full width at half-maximum of the diffraction peak. The calculated results of $TiO_2$ NPs are ~5, 10, 18, 25, 40 and 100 nm, respectively.

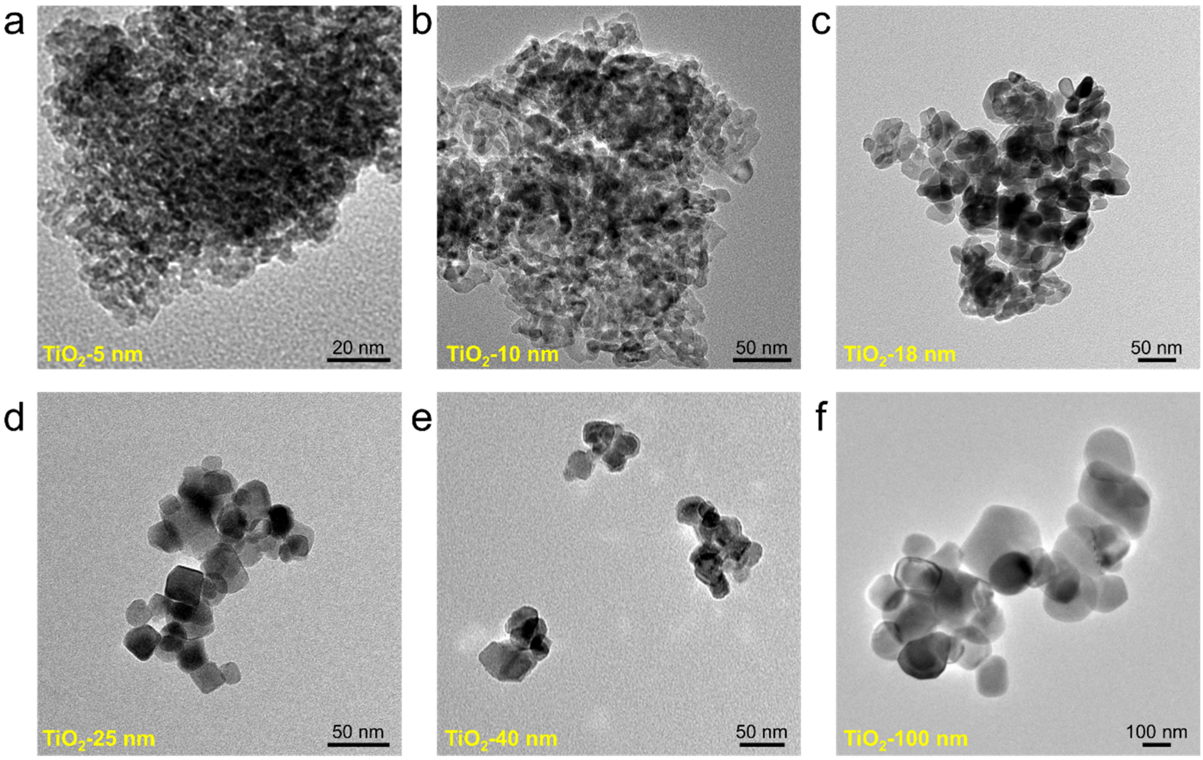


**Supplementary Figure 2.** TEM images of $TiO_2$ NPs. (a) $TiO_2$-5 nm, (b) $TiO_2$-10 nm, (c) $TiO_2$-18 nm, (d) $TiO_2$-25 nm, (e) $TiO_2$-40 nm, and (f) $TiO_2$-100 nm.

**Supplementary Table 1**. Morphology of different $TiO_2$ NPs.

| Sample | NP's size calculated based on Scherrer formula | BET specific surface area (SSA) | NP's size calculated based on SSA |
|---|---|---|---|
| $TiO_2$-5 nm | 4.96 nm | 303 $m^2$ $g^{-1}$ | 5.18 nm |
| $TiO_2$-10 nm | 9.86 nm | 133 $m^2$ $g^{-1}$ | 11.81 nm |
| $TiO_2$-18 nm | 17.93 nm | 80 $m^2$ $g^{-1}$ | 19.63 nm |
| $TiO_2$-25 nm | 24.68 nm | 57 $m^2$ $g^{-1}$ | 27.55 nm |
| $TiO_2$-40 nm | 39.82 nm | 40 $m^2$ $g^{-1}$ | 39.27 nm |
| $TiO_2$-100 nm | 98.23 nm | 11 $m^2$ $g^{-1}$ | 142.79 nm |

**Supplementary Section 2.** Electrochemical sodium-ion storage of $TiO_2$ NPs.

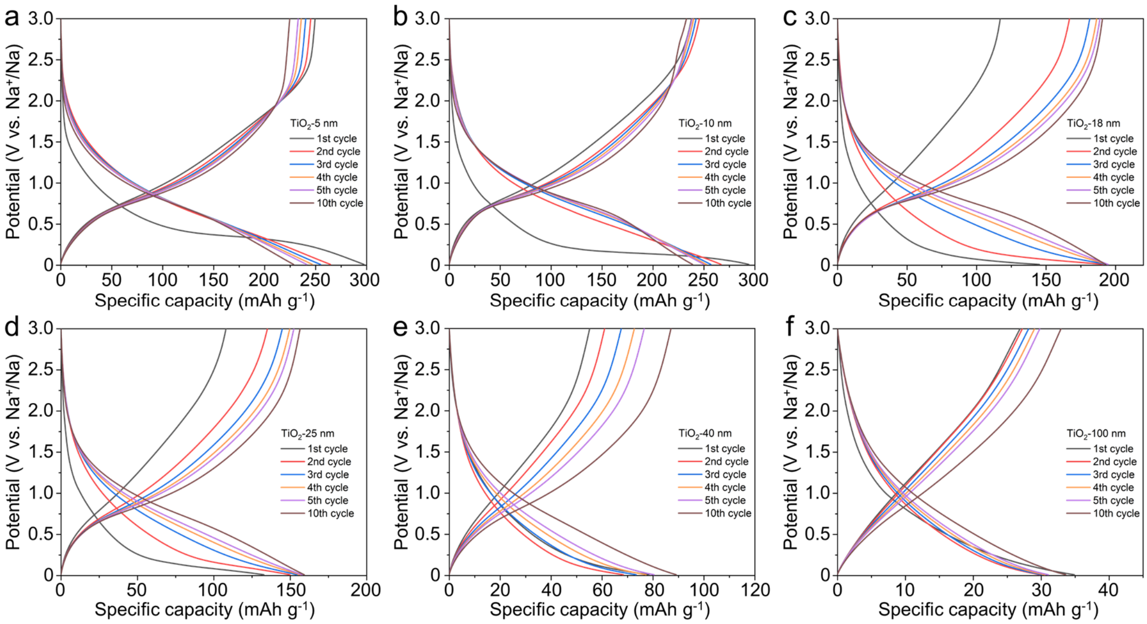


**Supplementary Figure 3.** Charge and discharge curves of the $TiO_2$ NPs at 0.1 A $g^{-1}$: (a) $TiO_2$-5 nm, (b) $TiO_2$-10 nm, (c) $TiO_2$-18 nm, (d) $TiO_2$-25 nm, (e) $TiO_2$-40 nm, and (f) $TiO_2$-100 nm.

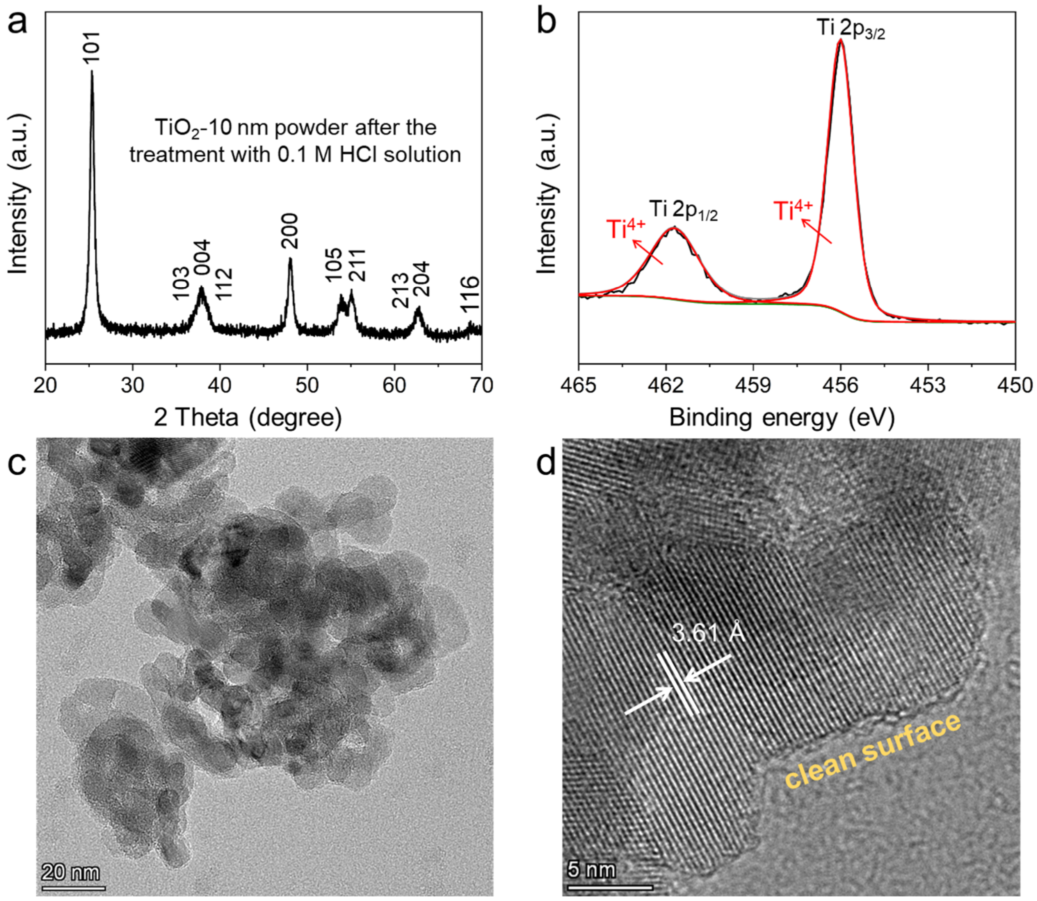


**Supplementary Figure 4**. XRD pattern (a), Ti 2p spectra (b), TEM image (c) and HRTEM image (d) of $TiO_2$-10 nm after the treatment with 0.1 M HCl solution. After soaking in 0.1 M HCl solution, the $TiO_2$-10 nm keeps in anatase phase and its surface remains the valance of $Ti^{4+}$, indicating the acid treatment has no effects on the $TiO_2$ NPs.

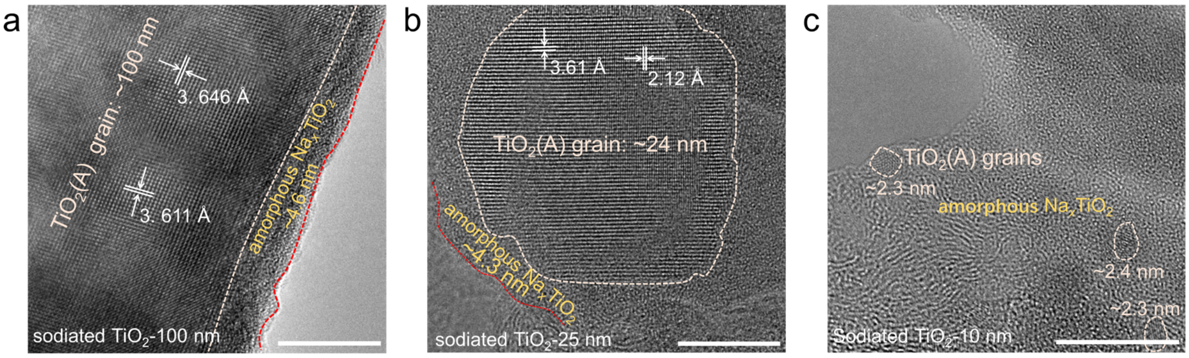


**Supplementary Figure 5**. The *ex-situ* HRTEM images of $TiO_2$-100 nm (a), $TiO_2$-25 nm (b) and $TiO_2$-10 nm (c) in the fully sodiated state after acid treatment. Scale bar: 10 nm. The amorphous layers are still observed after removing the SEI layers (acid treatment), confirming the amorphous transition is caused by the electrochemically sodiation reaction.

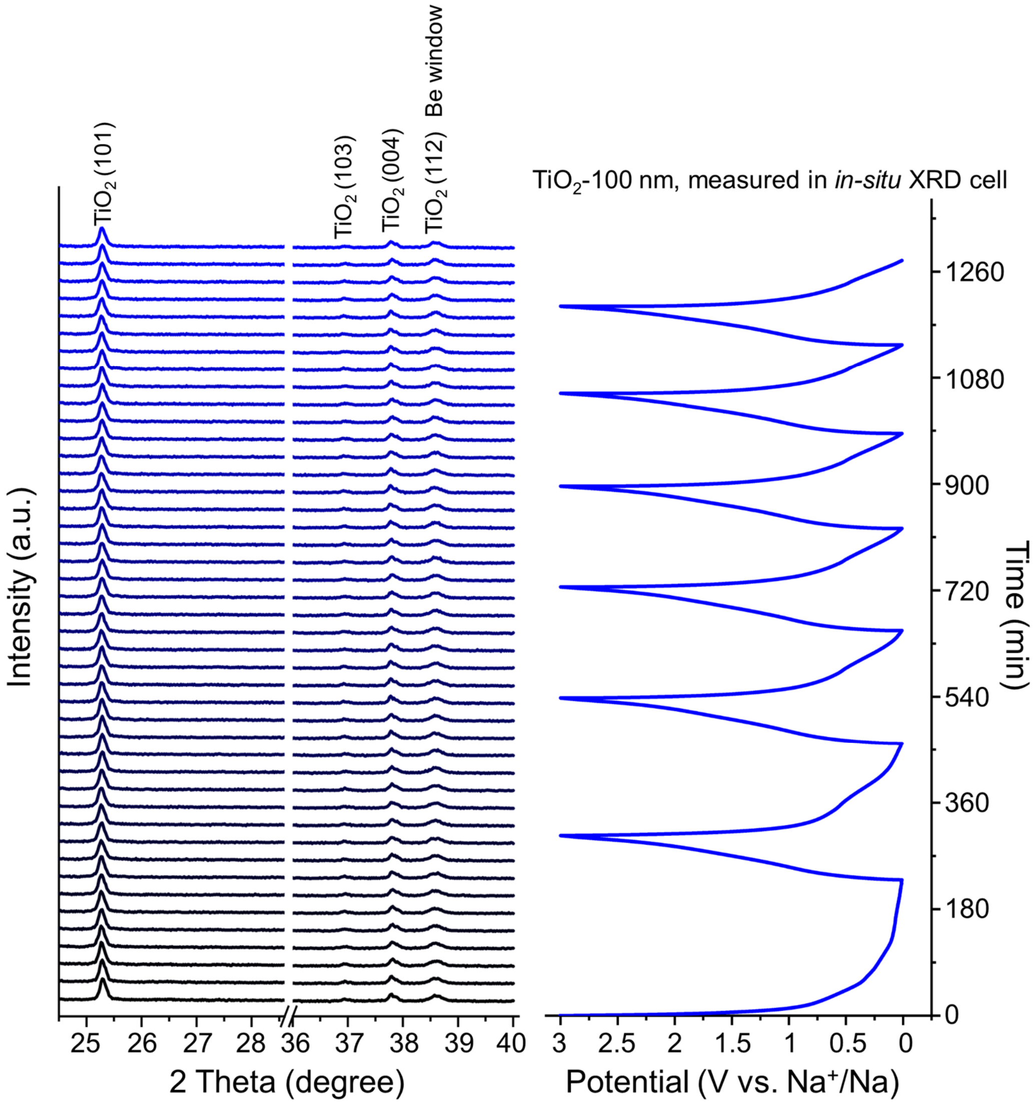


**Supplementary Figure 6.** *In-situ* XRD patterns of $TiO_2$-100 nm. The electrochemical performance was measured using an in-situ XRD cell using Be foil as both the X-ray transparent window and current collector. As a result of this cell design, side reactions and electrolyte decomposition were more pronounced, leading to the longer initial sodiation curve when compared to the electrochemical performance measured in coin cell (Supplementary Figure 3f).

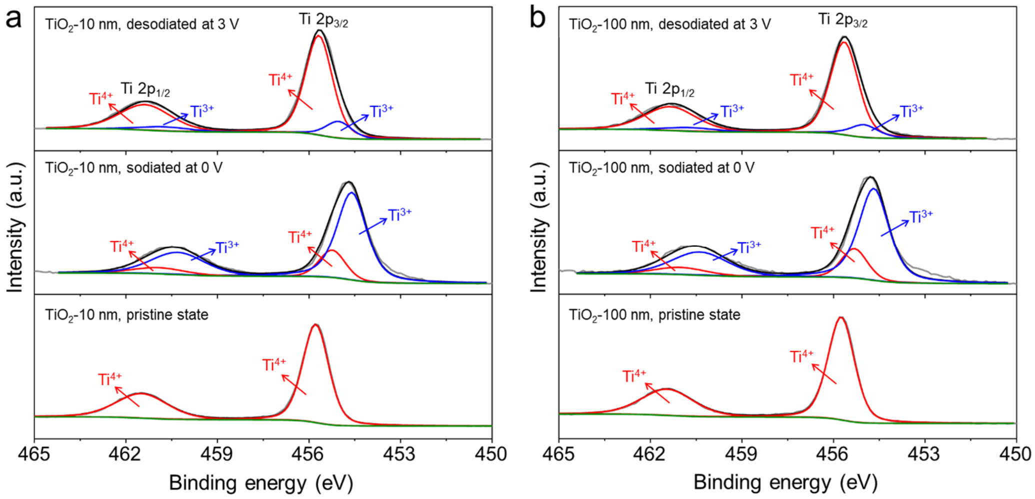


**Supplementary Figure 7.** Ex-situ XPS spectra analysis. (a) Ti 2p spectra of the $TiO_2$-10 nm electrode at different states. (b) Ti 2p spectra of the $TiO_2$-100 nm electrode at different states. Based on the fitting results (Supplementary Table 2), it is found that both materials exhibit the presence of the same amorphous surface composition ($Na_xTiO_2$ with $x$ = 0.8).

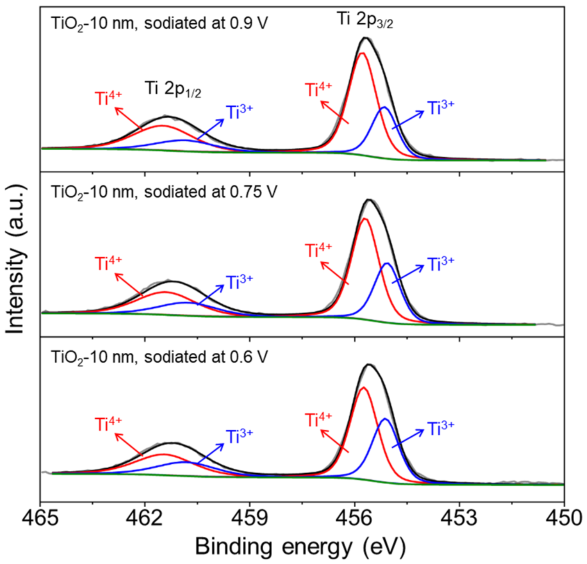


**Supplementary Figure 8.** Ti 2p spectra of the $TiO_2$-10 nm electrode at potentials of 0.9 V, 0.75 V and 0.6 V *vs.* $Na^+$/Na, after 3 cycles. According to Supplementary Table 2, it is found that the sodiation peak at ~0.75 V is mainly based on the redox of $Ti^{4+}/Ti^{3+}$.

**Supplementary Table 2.** Analysis of *ex-situ* XPS peaks for the $TiO_2$-10 nm and $TiO_2$-100 nm. The peak separation between Ti $2p_{3/2}$ and Ti $2p_{1/2}$ is 5.7 eV and the areas of the peaks are in the ratio of 2:1.

| Sample | Position | Ti $2p_{3/2}$ (4+) | Ti $2p_{1/2}$ (4+) | Ti $2p_{3/2}$ (3+) | Ti $2p_{1/2}$ (3+) |
|---|---|---|---|---|---|
| | (eV) | 455.8 ± 0.2 | 461.5 ± 0.2 | 455.1 ± 0.2 | 460.8± 0.2 |
| $TiO_2$ 10 nm, pristine state | area | 96818 | 48409 | -- | -- |
| | percentage | 100% | | -- | |
| $TiO_2$ 10 nm, sodiated at 0.01 V | area | 5466 | 2733 | 22904 | 11452 |
| | percentage | 19.3% | | 80.7% | |
| $TiO_2$-10 nm, desodiated at 3 V | area | 53980 | 26990 | 8590 | 4295 |
| | percentage | 86.3% | | 13.7% | |
| $TiO_2$-100 nm, pristine state | area | 55720 | 27860 | -- | -- |
| | percentage | 100% | | -- | |
| $TiO_2$-100 nm, sodiated at 0.01 V | area | 1996 | 998 | 8114 | 4057 |
| | percentage | 19.7% | | 80.3% | |
| $TiO_2$-100 nm, desodiated at 3 V | area | 17670 | 8835 | 2650 | 1325 |
| | percentage | 87.0% | | 13.0% | |
| $TiO_2$-10 nm, sodiated at 0.9 V | area | 22256 | 11128 | 9835 | 4917.5 |
| | percentage | 69.4% | | 30.6% | |
| $TiO_2$-10 nm, sodiated at 0.75 V | area | 20796 | 10398 | 11444 | 5722 |
| | percentage | 64.5% | | 35.5% | |
| $TiO_2$-10 nm, sodiated at 0.6 V | area | 19322 | 9661 | 12940 | 6470 |
| | percentage | 59.9% | | 40.1% | |

**Supplementary Table 3.** The specific capacity of the $TiO_2$ NPs (this work) and previously reported $TiO_2$ with different particle sizes.

| Sample | Particle size (nm) | Specific surface area ($m^2$ $g^{-1}$) | Specific capacity (mAh $g^{-1}$) | Testing conductions | Electrode composite |
|---|---|---|---|---|---|
| $TiO_2$-5 nm [this work] | 4.96* | 303 | 265 | at 100 mA $g^{-1}$ in 0.01-3 V | $TiO_2$:KJB:CMC:SBR =85:7:4:4 |
| $TiO_2$-10 nm [this work] | 10.21* | 133 | 264 | | |
| $TiO_2$-18 nm [this work] | 17.93* | 80 | 195 | | |
| $TiO_2$-25 nm [this work] | 24.68* | 57 | 161 | | |
| $TiO_2$-40 nm [this work] | 39.82* | 40 | 102 | | |
| $TiO_2$-100 nm [this work] | 98.23* | 11 | 40 | | |
| $TiO_2$(A) NPs (30 nm) [1] | 30 | -- | 150 | at 33.5 mA $g^{-1}$ in 0.1-2 V | $TiO_2$:Super C65:CMC =70:20:10 |
| $TiO_2$(A)@C NPs (~11 nm) [2] | 11 | 119.8 | 227 | at 33.5 mA $g^{-1}$ in 0.05-2 V | $TiO_2$:Super C65:PVdF =65:25:10 |
| $TiO_2$(A) NPs (<25 nm) [3] | <25 | -- | 192 | at 0.1 A $g^{-1}$ in 0.01-3 V | $TiO_2$:CB:PVdF =70:20:10 |
| $TiO_2$(A) NPs (22.5 nm) [4] | 22.5* | 80.72 | 188 | at 33.6 mA $g^{-1}$ in 0.01-3 V | $TiO_2$:SP:CMC =70:15:15 |
| TOC-80 [5] | 15* | 124 | 243 | at 84 mA $g^{-1}$ in 0.01-2.5 V | $TiO_2$:SP:CMC =70:15:15 |
| TOC-150 [5] | 19* | 76 | 223 | | |
| TOC-300 [5] | 22* | 61 | 187 | | |

* calculated based on Scherrer formula; CB: carbon black

**References**


1. Wu, L. *et al.* Unfolding the mechanism of sodium insertion in anatase $TiO_2$ nanoparticles. *Adv. Energy Mater.* **5**, 1401142 (2015).
2. Tahir, M. N. et al. Extraordinary performance of carbon-coated anatase TiO2 as sodium-ion anode. Adv. Energy Mater. 6, 1501489 (2016).
3. Xu, Z.-L. *et al.* Engineering solid electrolyte interphase for pseudocapacitive anatase $TiO_2$ anodes in sodium-ion batteries. *Adv. Funct. Mater.* **28**, 1802099 (2018).
4. Chen, J. *et al.* Black Anatase titania with ultrafast sodium-storage performances stimulated by oxygen vacancies. *ACS Appl. Mater. Inter.* **8**, 9142-9151 (2016).
5. Chen, J. *et al.* Size-tunable olive-like anatase $TiO_2$ coated with carbon as superior anode for sodium-ion batteries. *Small* **12**, 5554-5563 (2016).

**Supplementary Section 3.** Kinetics analysis for the sodium-ion storage of $TiO_2$ NPs.

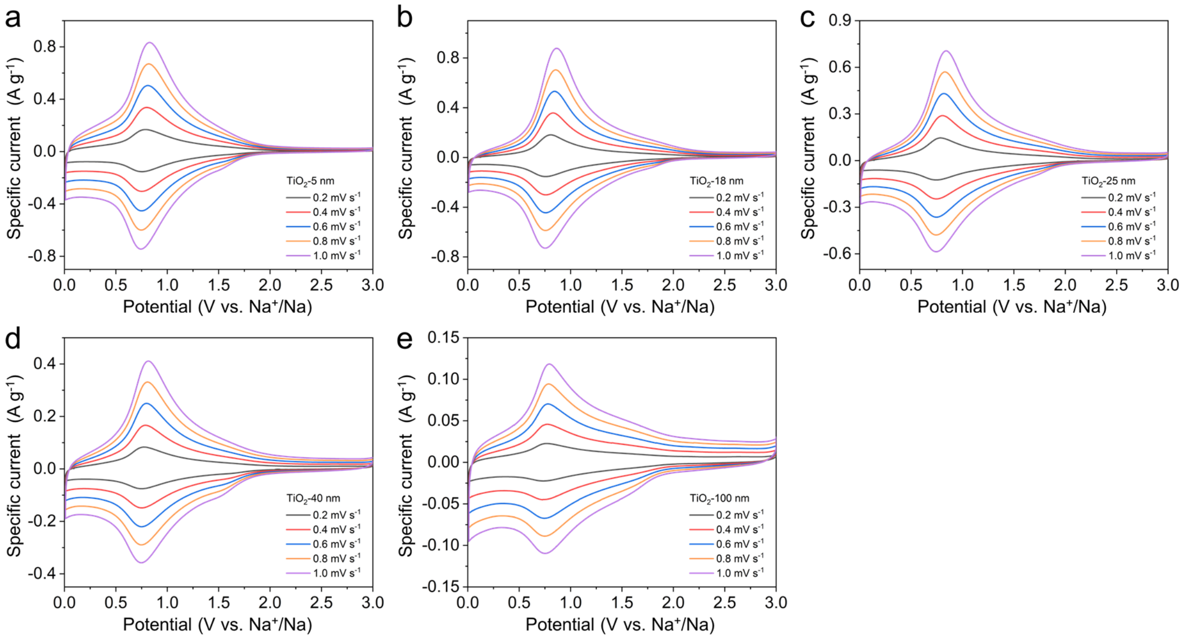


**Supplementary Figure 9.** CV curves for the sodium-ion storage of $TiO_2$-NPs at sweep rates from 0.2 to 1 mV $s^{-1}$: (a) $TiO_2$-5 nm, (b) $TiO_2$-18 nm, (c) $TiO_2$-25 nm, (d) $TiO_2$-40 nm, and (e) $TiO_2$-100 nm.

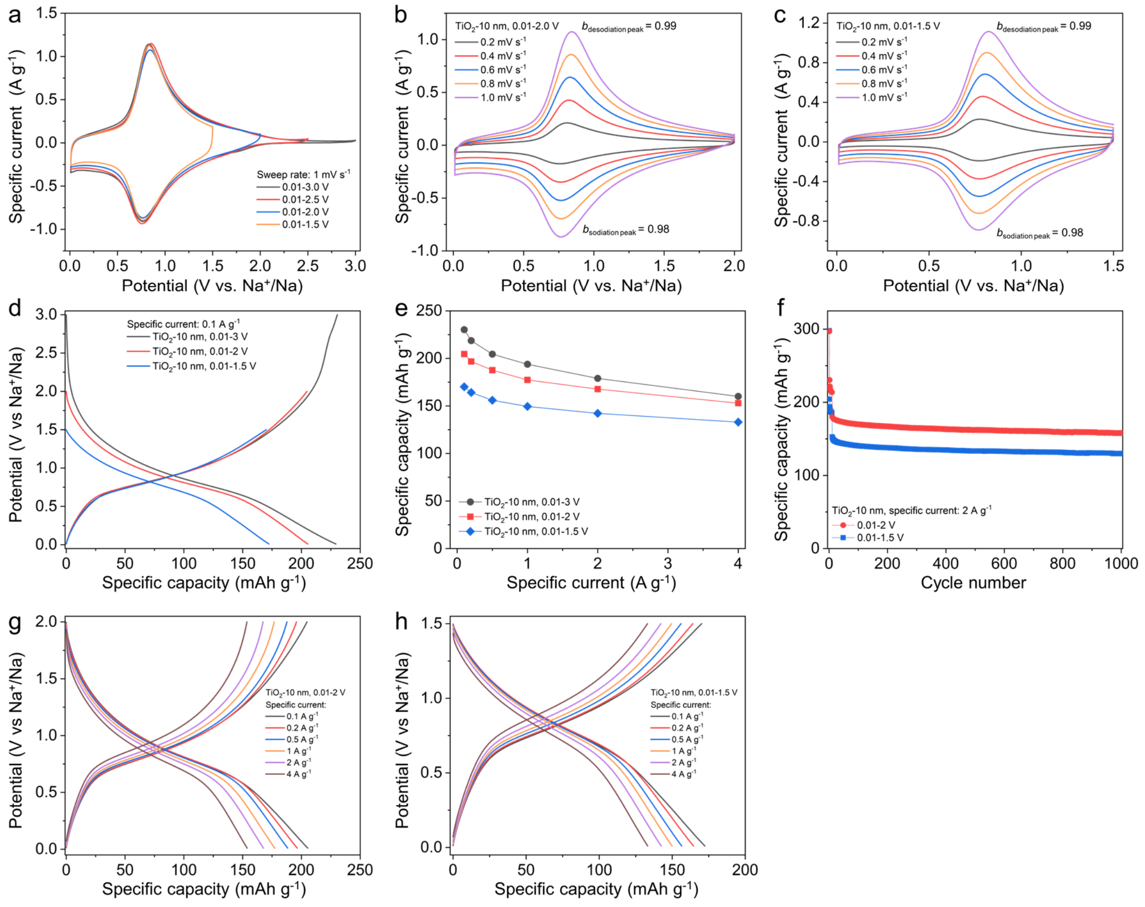


**Supplementary Figure 10**. (a) CV curves of $TiO_2$-10 nm in different potential windows, at 1 mV $s^{-1}$. The CV curves of $TiO_2$-10 nm in 0.01-2 V (b) and 0.01-1.5 V (c), at sweep rates ranging from 0.2 to 1.0 mV $s^{-1}$. (d) Charge and discharge curves of $TiO_2$-10 nm in different potential windows at 0.1 A $g^{-1}$. The rate capabilities (e) and cycling performance of $TiO_2$-10 nm at 2 A $g^{-1}$ (f) in different potential windows. Charge and discharge curves of $TiO_2$-10 nm in 0.01-2 V (g) and 0.01-1.5 V (h), respectively.

The $TiO_2$-10 nm anodes display the coupled redox peaks in different potential windows, indicating the highly reversible surface-redox reactions. According to the kinetics analysis, the $TiO_2$-10 nm anodes also exhibit surface-controlled kinetics in the range of 0.01-2.0 V (Supplementary Fig. 10b) and 0.01-1.5 V (Supplementary Fig. 10c). The charging and discharging curves show the reversible redox reaction in the different potential ranges (Supplementary Figure 10d). Meanwhile, the $TiO_2$-10 nm anodes showed excellent rate capability and cycling stability in the different potential ranges (Supplementary Figure 10e-h).

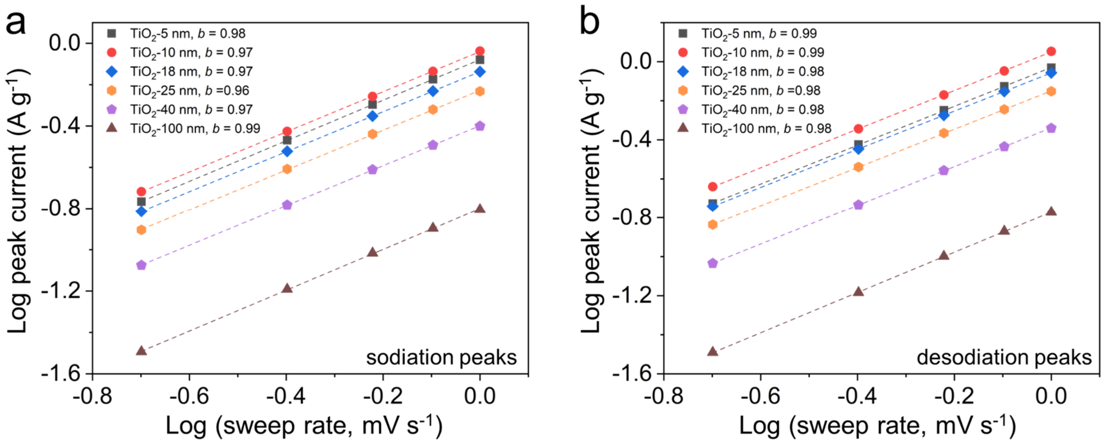


**Supplementary Figure 11**. *b*-value fitting of the sodiation peaks (a) and desodiation peaks (b) of the $TiO_2$ NPs, respectively.

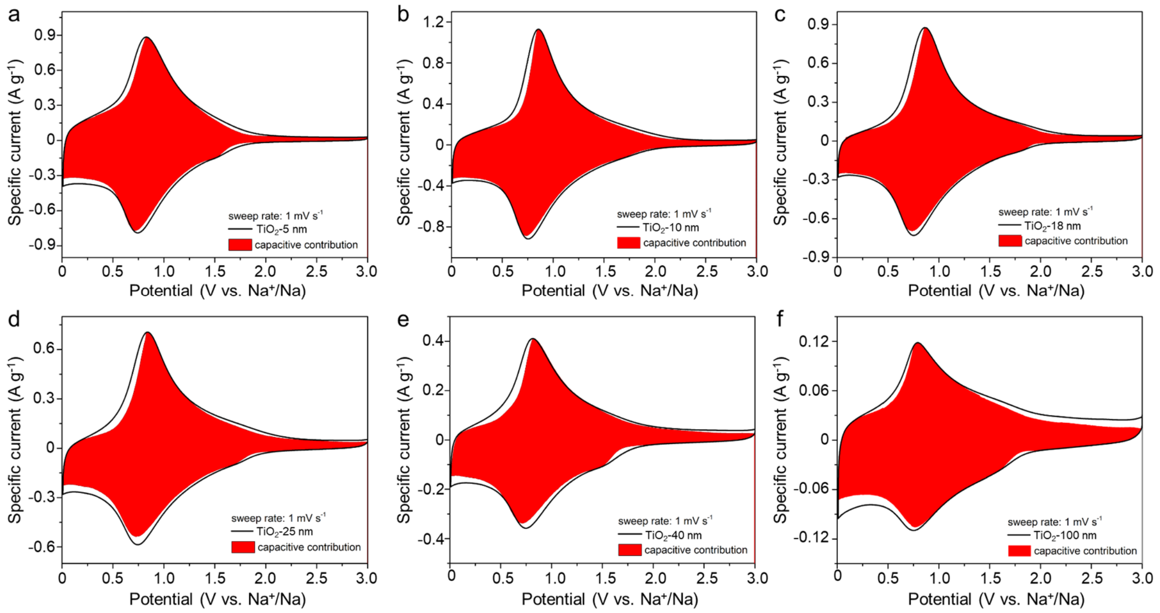


**Supplementary Figure 12**. Capacitive contributions to sodium-ion storage for $TiO_2$ NPs at 1.0 mV s$^{-1}$, (a) $TiO_2$-5 nm, (b) $TiO_2$-10 nm, (c) $TiO_2$-18 nm, (d) $TiO_2$-25 nm, (e) $TiO_2$-40 nm, and (f) $TiO_2$-100 nm.

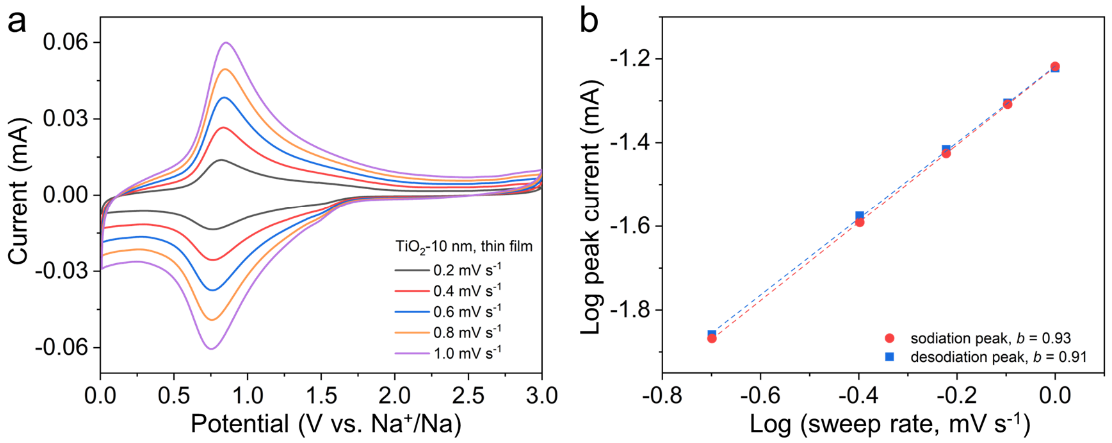


**Supplementary Figure 13**. (a) CV curves for the thin-film electrode of $TiO_2$-10 nm (100 μg $cm^{-2}$, without any binder or carbon additives), at sweep rates from 0.2 to 1 mV $s^{-1}$. (b) The corresponding *b*-value fitting of the sodiation and desodiation peaks for the thin-film electrode.

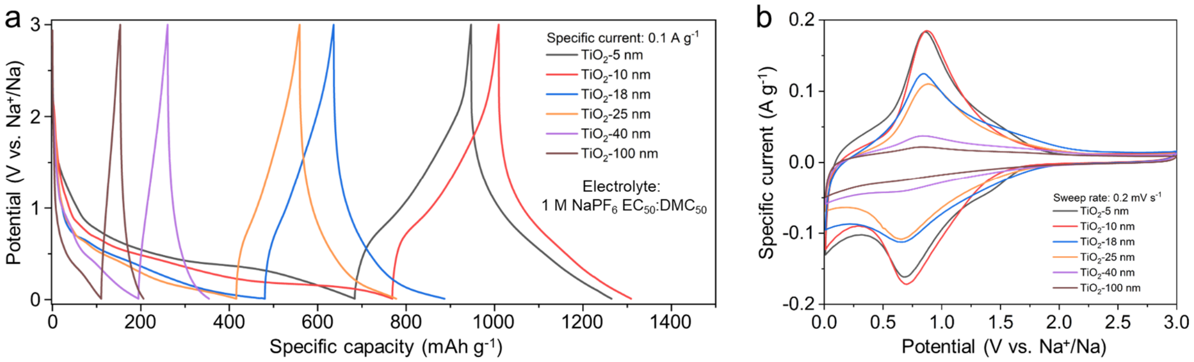


**Supplementary Figure 14**. The initial sodiation and desodiation curves at 0.1 A $g^{-1}$ (**a**) and CV curves at the sweep rate of 0.2 mV $s^{-1}$ (**b**) of different $TiO_2$ NPs measured in the electrolyte of 1 M $NaPF_6$ in $EC_{50}$:$DMC_{50}$. The initial discharge capacity of $TiO_2$ NPs in ester-based electrolyte (Supplementary Figure 13a) is higher than those in ether-based electrolyte (Figure 1a), owing to the serious decomposition of carbonate electrolyte and the formation of thick SEI layers. Moreover, the specific capacity of $TiO_2$ NPs in ester-based electrolyte is size-dependent as well. The CV curves of the $TiO_2$ NPs measured in ester-based electrolyte (Supplementary Figure 13b) show a similar shape and redox reaction potential compared to those in ether-based electrolyte (Figure 4a). These results indicate that the electrolyte system does not affect the surface-redox sodium-ion storage mechanism of anatase. However, the as-formed thick SEI layers from the ester-based electrolyte (1 M $NaPF_6$ in $EC_{50}$:$DMC_{50}$) limit the rate performance.

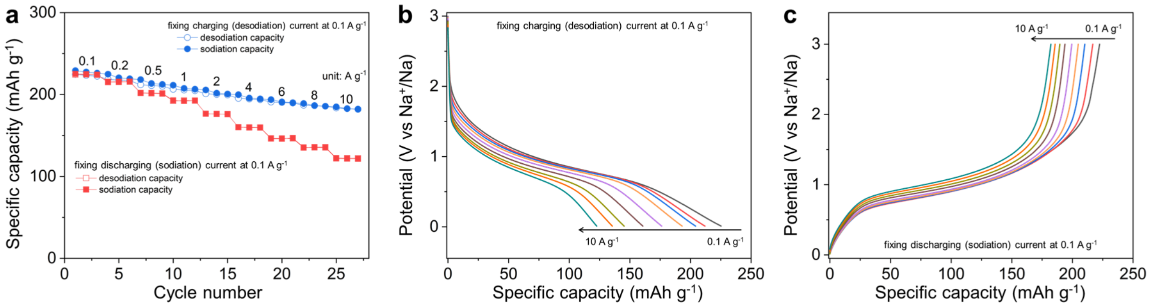


**Supplementary Figure 15**. (a) Capacity plots with different discharge (sodiation) and charge (desodiation) currents. (b) The discharge curves of $TiO_2$-10 nm though fixing the charge (desodiation) current at 0.1 A $g^{-1}$. (c) The charge curves of $TiO_2$-10 nm though fixing the discharge (desodiation) current at 0.1 A $g^{-1}$.

When the desodaition current is fixed at 0.1 A $g^{-1}$, the $TiO_2$-10 nm anode exhibits fast sodiation performance (Supplementary Figure 15b): a high capacity of 160 mAh $g^{-1}$ at 4 A $g^{-1}$ and 121 mAh $g^{-1}$ at 10 A $g^{-1}$ (corresponding to a time of 44 seconds to reach 54% of total capacity). Furthermore, when the sodiation current is fixed at 0.1 A $g^{-1}$ (close to full storage of $Na^+$ in $TiO_2$), the $TiO_2$-10 nm anode delivers high desodiation capacity at high rates (Supplementary Fig. 15c), *e.g.* a high capacity of 182 mAh $g^{-1}$ at 10 A $g^{-1}$, showing the excellent high-rate ability. Comparing the two results in Supplementary Figure 15a and b, the stored capacity of $TiO_2$ is dependent on the sodiation rates.

**Supplementary Section 4.** Kinetics analysis for the lithium-ion storage of $TiO_2$ NPs.

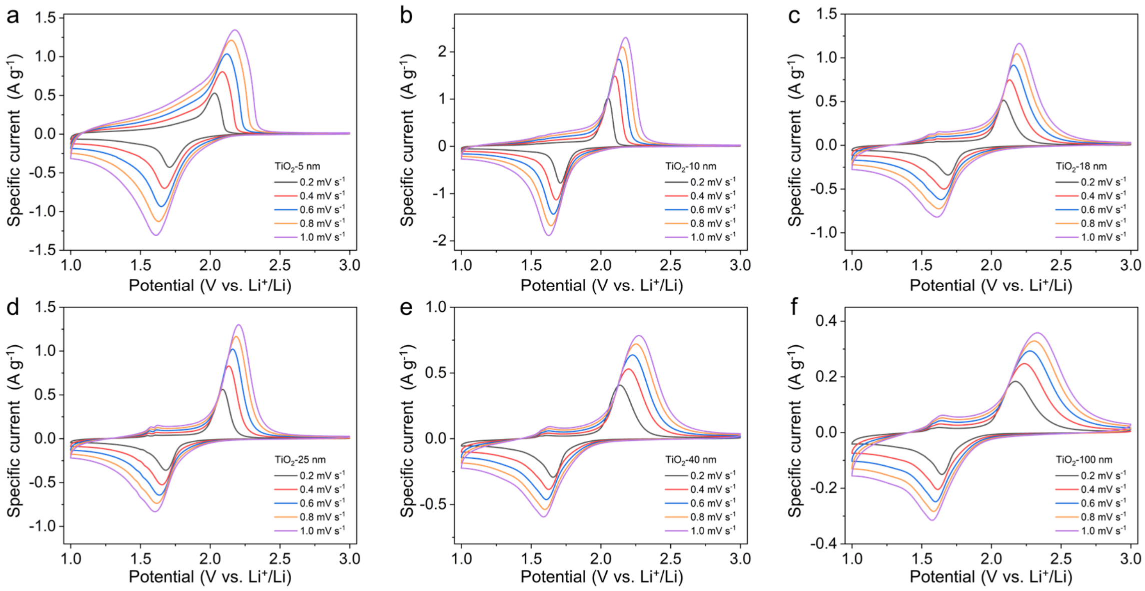


**Supplementary Figure 16.** The CV curves for the lithium-ion storage of $TiO_2$ NPs at sweep rates from 0.2 to 1 mV $s^{-1}$: (a) $TiO_2$-5 nm, (b) $TiO_2$-10 nm, (c) $TiO_2$-18 nm, (d) $TiO_2$-25 nm, (e) $TiO_2$-40 nm, and (f) $TiO_2$-100 nm.

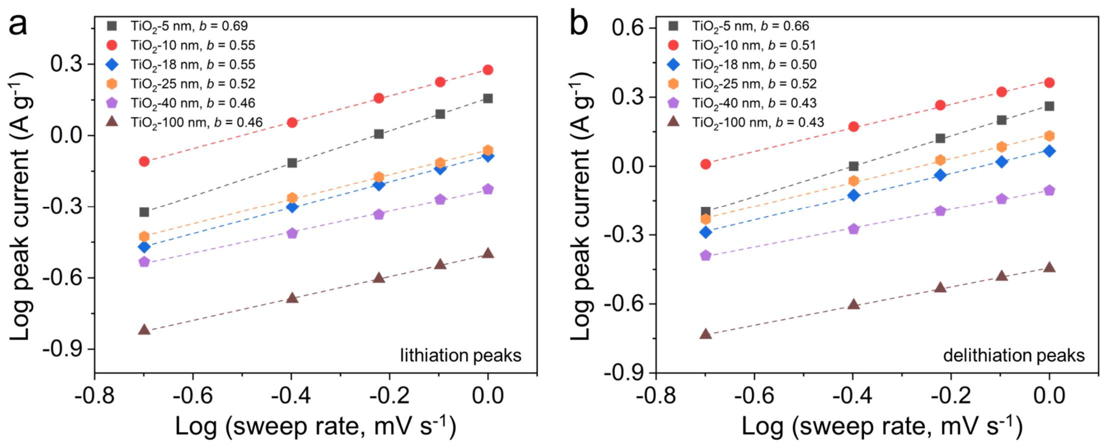


**Supplementary Figure 17**. The *b*-value fitting of the lithiation peaks (a) and delithiation peaks (b) of the $TiO_2$ NPs, respectively.

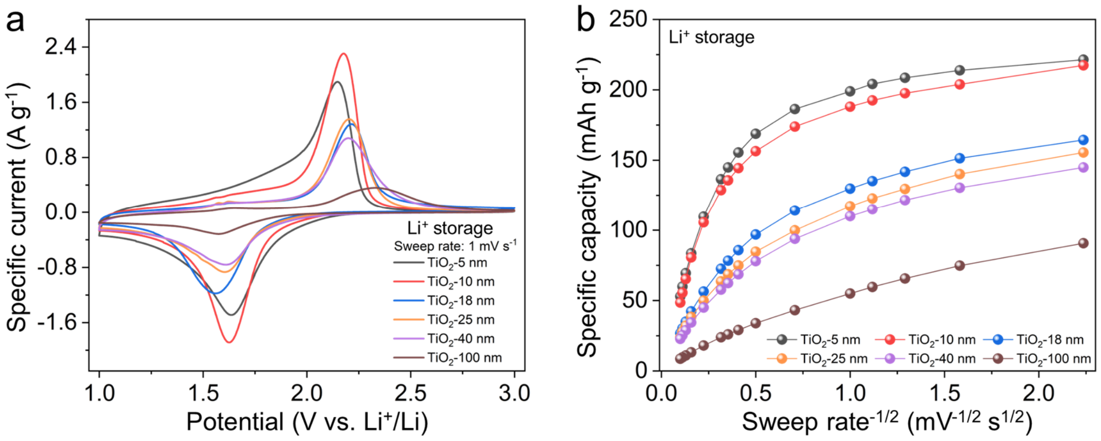


**Supplementary Figure 18.** (a) The CV curves of the different $TiO_2$ NPs for lithium-ion storage at a sweep rate of 1 mV $s^{-1}$. (b) Specific capacity *vs.* sweep rate$^{-1/2}$ curves of the $TiO_2$ NPs.

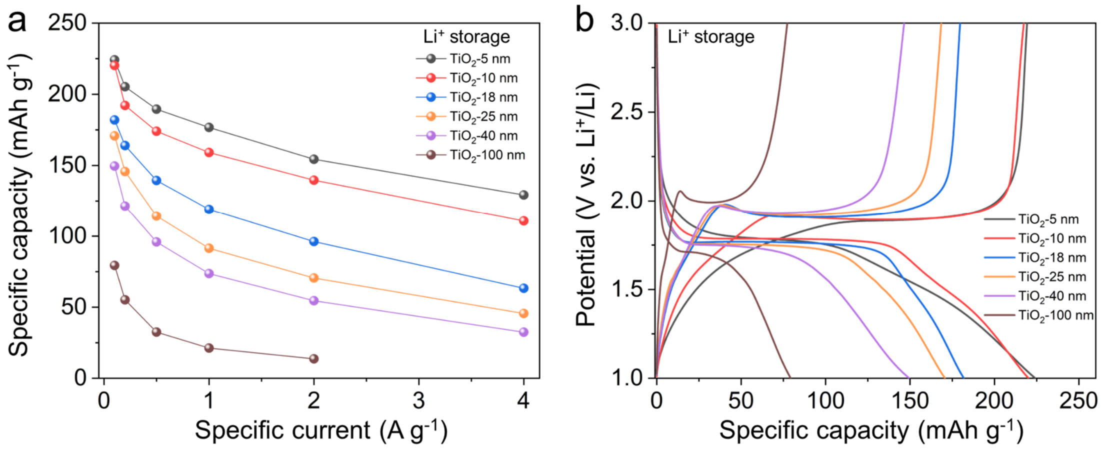


**Supplementary Figure 19.** (a) The rate capability of the different $TiO_2$ NPs for lithium-ion storage. (b) The corresponding charge and discharge curves of the $TiO_2$ NPs at a specific current of 0.1 A $g^{-1}$.
